# Human chaperone DNAJB6b suppresses tau fibril formation through co-aggregation

**DOI:** 10.1038/s42004-026-02108-1

**Published:** 2026-08-01

**Authors:** Andreas Carlsson, Emil Axell, Johan Wallerstein, Dev Thacker, Ulf Olsson, Sara Linse

**Affiliations:** 1 Biochemistry and Structural Biology, Lund University, Lund, Sweden; 2 Physical Chemistry, Lund University, Lund, Sweden

**Keywords:** Biophysical chemistry, Chaperones, Protein aggregation

## Abstract

The aggregation of the tau protein into intraneuronal fibrillar tangles is closely associated with the pathology of Alzheimer’s disease. The endogenous defense system against this process includes molecular chaperones, among which DNAJB6b has emerged as a key component. Using a tau model system comprising the tau fragment 304-380C322S, which spans the amyloidogenic core of ex vivo Alzheimer’s disease fibrils, we investigated the impact of DNAJB6b on tau fibril formation. Here, we show that DNAJB6b potently delays tau aggregation by co-assembling with small tau aggregates and by binding to mature fibrils, thereby reducing their ability to catalyze further fibril growth. This interplay between tau and the chaperone results in greatly reduced fibril formation rate and a lower final fibril mass, which we interpret as increased tau solubility. Moreover, solution-state NMR spectroscopy confirms that DNAJB6b does not interact with tau monomers.

## Abstract (web summary)

Human chaperone DNAJB6b has emerged as a key component involved in the endogeneous defense system against tau protein aggregation in the context of Alzheimer’s disease. Here, the authors study the impact of DNAJB6b on tau fibril formation, showing that it delays tau aggregation by co-assembling with small tau aggregates and by binding to mature fibrils, reducing their ability to catalyze further fibril growth.

## Introduction

Alzheimer’s disease (AD) is characterized by progressive memory loss and personality changes. On the molecular level, AD is associated with the aggregation of tau protein and amyloid *β* peptide into highly ordered fibrillar structures that accumulate intra- and extracellularly of neurons, respectively. In the case of tau, the fibrils consist of several isoforms and are found in multiple neurodegenerative disorders collectively known as tauopathies^1^. AD, the most prevalent tauopathy, affects millions of people worldwide, with incidence expected to continue rising^2^. Tau is the major component in neurofibrillary tangles in AD-affected brains^3^.

To prevent harmful precipitation of tau and other proteins, cells rely on a native defense system provided by molecular chaperones. These proteins constitute a central part of the proteostasis network, ensuring proper protein folding and directing misfolded species toward refolding or degradation pathways^4^. During disease progression, this system becomes compromised and can no longer maintain proteostasis^5^. Among molecular chaperones, DNAJB6b (hereafter referred to as JB6) has attracted particular attention due to its exceptional potency in suppressing amyloid formation. In a cell-line screening study with 49 chaperones, JB6 emerged as the most potent suppressor of tau aggregation^6^. Another study reports that JB6 was most potent in preventing tau aggregation when comparing different J-domain proteins^7^.

In the cell, JB6 functions together with Hsp70. The binding of clients by JB6 facilitates their processing by the Hsp70 chaperone machinery^8^. Notably, even in the absence of ATP or HSP70, JB6 exhibits strong inhibitory effects on amyloid formation. The amyloid-regulatory effects of JB6 (both in the absence and presence of co-factors) extend to a wide range of amyloidogenic proteins and peptides implicated in various neurodegenerative and metabolic diseases. These include polyglutamine peptides (Huntington’s disease)^9–14^, amyloid *β* peptides (AD)^12,15–17^, tau (AD)^6,7^, *α*-synuclein (Parkinson’s disease and other synucleinopathies)^18–21^, TDP43 (ALS)^22^, and FUS (ALS/frontotemporal dementia)^23^.

In addition to retarding amyloid formation, JB6 has been shown to enhance the apparent solubility of client proteins. Such an effect has been documented in vitro for amyloid *β*^15^ and *α*-synuclein^21^. The solubility increase is proposed to rely on the formation of co-aggregates between JB6 and its client proteins^24^, and such co-aggregates have indeed been observed for JB6 and *α*-synuclein^21^.

JB6 itself has a strong tendency to form oligomers with the physicochemical characteristics of micelles^12,25–28^. Full chaperone activity requires dissociation of these oligomers into subunits^17^, which are monomers^25^.

Several lines of evidence point to a role of JB6 in preventing tauopathies. The expression levels of JB6 are downregulated in the brains of individuals suffering from progressive supranuclear palsy^29^, a primary tauopathy characterized by the accumulation of insoluble tau. Moreover, JB6 has been shown to modulate the aggregation of tau in AD cell models. Overexpression of the chaperone reduces the amount of precipitated tau, whereas knockdown of JB6 led to increased tau aggregation^6,7^. These findings support a protective role of JB6 in maintaining tau proteostasis. However, the molecular mechanisms by which JB6 exerts its inhibitory effects on tau aggregation—particularly independent of Hsp70—remain poorly understood.

In the present study, we address several key questions: Does JB6 by itself influence tau aggregation, and if so, does it affect the kinetics, the equilibrium, or both? In the case of observable effects, which of the microscopic steps in the tau self-assembly pathway are affected by the presence of JB6? What are the affinities of JB6 for tau fibrils and monomers, respectively? Are different types of aggregates formed when JB6 is added to tau monomers before aggregation and present during fibril formation, compared to when JB6 is added afterward to pre-formed fibrils?

**Fig. 1:**
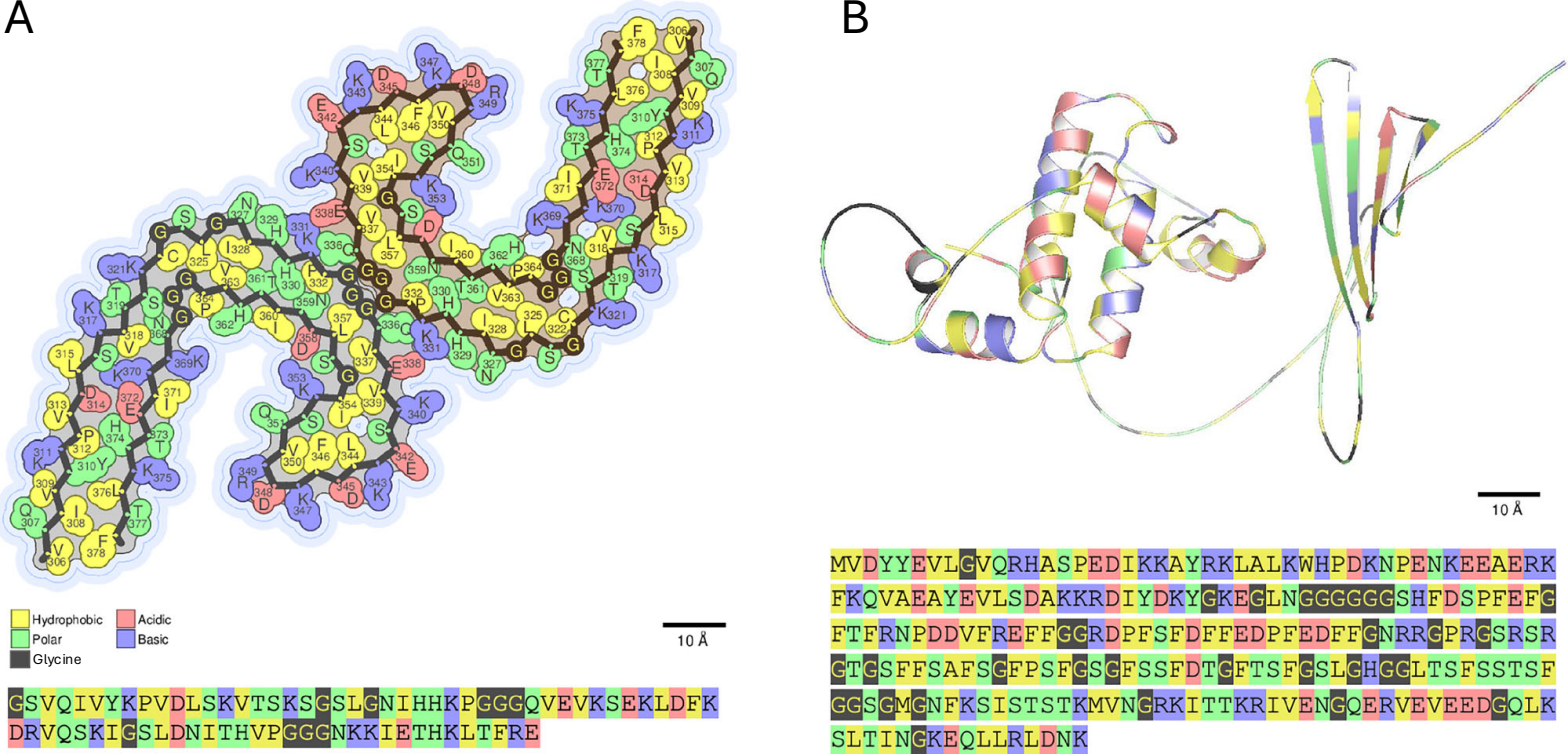
Structure and sequence of tau and JB6. **A** Paired helical filament structure of ex vivo tau fibrils from AD patients, PDB: 5O3L^31^, encompassing residues 306-378, visualized using Amyloid Illustrator^32^, showing one plane of the filament cross-section. The sequence of the tau fragment used in this work (304-380C322S) is given below the structure. **B** DNAJB6b structure prediction by AlphaFold2^33,34^ and its sequence.

To answer these questions, we measured tau aggregation kinetics under varying degrees of seeding and across a range of JB6 concentrations. We employed our previously developed tau model system^30^ (residues 304-380C322S), which spans the amyloidogenic core of ex vivo AD fibrils^31^. The cysteine-to-serine mutation enables the fragment to be studied under non-reducing conditions without the complication of disulfide bonding. Figure 1 shows sequence and structure representations of the tau fragment used (PDB: 5O3L,^31^, visualized using Amyloid Illustrator^32^) and JB6, using a structure prediction by AlphaFold2^33,34^. This fragment of tau readily aggregates in vitro without the need for external inducers, shaking, or extreme buffer conditions^30^, providing a robust platform for dissecting the effects of inhibitors on specific microscopic steps of amyloid formation. Kinetic assays were complemented by quantitative analyses of JB6 and tau concentrations in both the supernatant and pelletable fractions throughout the aggregation time course, using HPLC with absorbance measurements^35^. Nuclear magnetic resonance (NMR) spectroscopy was employed to probe potential interactions between monomeric tau and JB6, while cryogenic electron microscopy (cryo-EM) was used to visualize and compare tau aggregates formed in the absence and presence of JB6.

We find that JB6 exhibits a remarkable potency in suppressing tau aggregation, acting substoichiometrically and in the nanomolar range. By analyzing the results of seeded and non-seeded kinetics assays using established aggregation models, we find that JB6 suppresses both primary and secondary growth processes of tau aggregation. We find that JB6 has no detectable affinity for tau monomers, but binds strongly to tau fibrils. Furthermore, we demonstrate that JB6 increases the apparent solubility of tau. Together, these findings reveal a multifaceted mechanism by which JB6 modulates both the kinetics and thermodynamics of tau aggregation, thereby providing the proteostasis network with necessary relief to maintain protein homeostasis. This mechanism provides a potential avenue for therapeutic intervention.

## Results

### Inhibition of tau aggregation by JB6

**Fig. 2:**
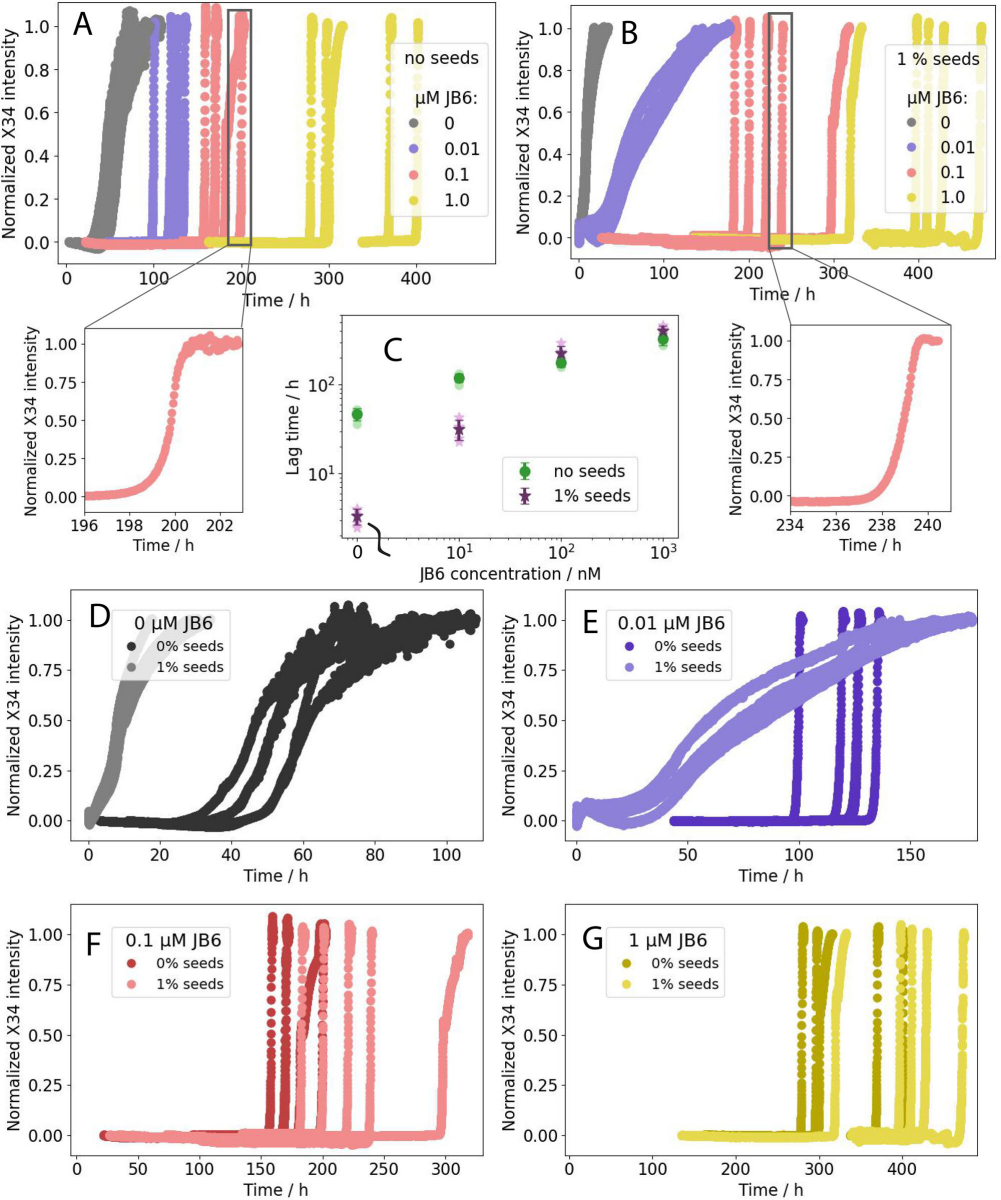
Aggregation kinetics of 7 μM tau at different JB6 concentrations. Five replicates. **A** Non-seeded reactions. **B** 1% seeded reactions. One of the kinetic traces in each panel A and B is shown with an expanded time-axis to clearly display the sigmoidal shape. The conditions were 37 °C, 20 mM sodium phosphate (NaP), 0.2 mM EDTA and 150 mM NaCl, pH 7.4, using the fluorescence intensity of the fibril-binding dye X34 as a reporter of fibril mass. **C** Lag times of all kinetic traces in (**A**) and (**B**), defined here as the time at which 10 % of the plateau value is reached. The mean value and standard deviation from *n* = 5 biologically independent samples are shown, with individual data points plotted in pale colors. **D–G** Replotted kinetic traces from (**A**) and (**B**), but with the same JB6 concentration in each panel, comparing data from non-seeded and 1% seeded samples.

To examine whether JB6 alone affects tau amyloid formation, aggregation kinetic studies were performed with tau in the presence of different JB6 concentrations, shown in Fig. 2, with no seeds in panel A, and in the presence of 1% pre-formed tau fibrils as seeds in panel B. It is evident from these kinetic traces that JB6 has a large retarding effect on tau aggregation, both in the absence and presence of 1% seeds. The aggregation lag phase is prolonged by JB6 in a concentration-dependent manner. Since the lag phase is much longer than the growth phase in the presence of JB6, the curve shape becomes difficult to resolve when the whole time course is plotted. Therefore, each panel includes a representative kinetic trace with an expanded time axis, in which the sigmoidal shape becomes clear. The retardation can also be seen by plotting the lag times versus JB6 concentration, Fig. 2C. The lag times are extracted as the time point at which 10 % of the plateau intensity is reached.

The kinetic traces are re-plotted per JB6 concentration to further visualize the effect on tau aggregation with and without 1% tau seeds (Fig. 2D–G). Seeding speeds up tau aggregation kinetics dramatically both in the absence and presence of JB6 at low JB6 concentrations (0.01 μM) (Fig. 2D, E). In contrast, at higher JB6 concentrations (0.1 and 1.0 μM), the chaperone appears to make the seeds completely inactive (Fig. 2F, G). For raw fluorescence data of the kinetic traces, see supplementary information, Fig. S1.

#### Kinetic analyses - effects on discrete microscopic steps

**Fig. 3:**
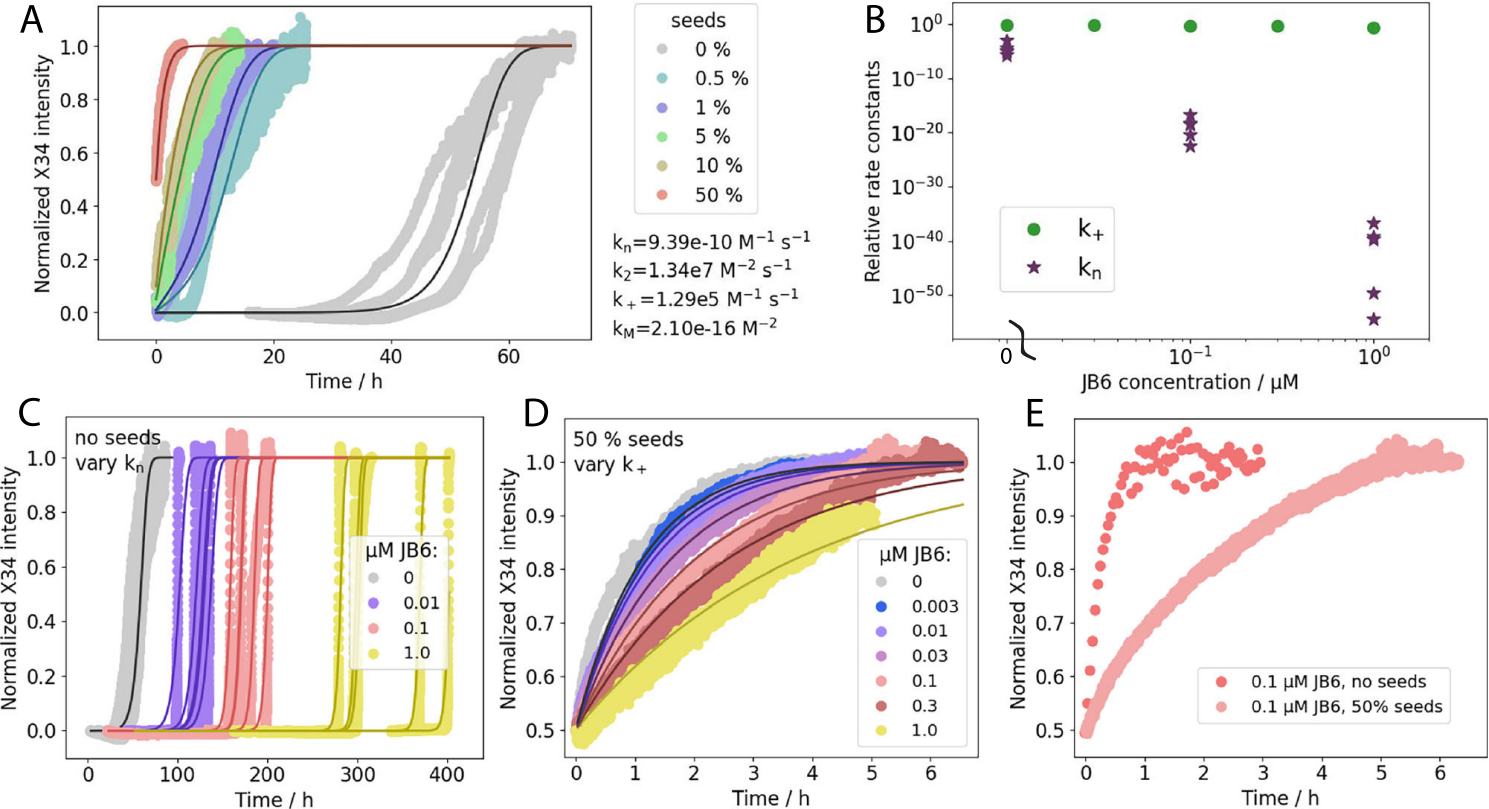
Fits of the tau aggregation traces to examine which microscopic steps JB6 affects. **A** Tau aggregation kinetics at varying seed concentrations. The total tau concentration was maintained at 7 μM in all samples except for the 50 % seed condition, which had 6.6 μM. AmyloFit^36^ was used to fit the data with a multi-step secondary nucleation dominated model with global fitting of tau rate constants and the saturation parameter k_M_, given in the lower right. **B** The change in elongation and primary nucleation rate constants with JB6 concentration, relative to the rate constants of tau alone. **C** Fits to the data from non-seeded experiments from Fig. 2A, with only k_n_ as the fitted parameter, keeping the other parameters fixed at the values determined for tau alone. **D** Fits of 50 % seeded reactions, with only k_+_ as fitted parameter, keeping the other parameters as determined for tau alone. **E** Comparing the last 50% of the curve of non-seeded reactions at 0.1 μM JB6, with the corresponding 50% seeded reaction.

Having established that JB6 has a strong retarding effect on the aggregation of tau, we next ask to what extent different microscopic steps of amyloid formation are affected. We attempt to answer this question by fitting models—which theoretically describe the fibril formation^36^—to aggregation kinetics data for tau with various concentrations of JB6 and at different seeding conditions. Using the rate constants of tau alone and varying one rate constant at a time in a JB6 concentration-dependent manner, we can test whether the decrease of one rate constant alone can describe the retardation by JB6. To do this, we first need well-determined rate constants of tau alone. Based on earlier work^30^, we use a multi-step secondary nucleation model to fit the aggregation kinetics. The rate constants of tau aggregation at the present condition (20 mM sodium phosphate (NaP), 0.2 mM EDTA, 150 mM NaCl, pH 7.4, 37 °C) are determined using various seed concentrations (Fig. 3A). The rate constants agree reasonably well with those earlier reported^30^, at a slightly different buffer composition. To constrain the fitting parameters, the concentration of fibril ends was estimated using cryo-EM imaging (Fig. S2). The average fibril length of the seeds was found to be 450 ± 200 nm (*n* = 25) and the fibril thickness profile can be described with two monomers per plane, consistent with findings in ref. ^31^. With an inter-plane distance of 4.7 Å, we estimate each seed fibril to consist of on average  ~ 2000 monomers.

In a monomer solution without seeds, primary nucleation is initially the rate-limiting step, since the nucleation barrier must be overcome before monomers can be consumed further through elongation and secondary nucleation. Decreasing the primary nucleation rate constant, k_n_, in a JB6 concentration-dependent manner describes the non-seeded data well (Fig. 3C). The decrease in k_n_ with increasing JB6 concentration is plotted in panel B.

With a similar approach, to isolate the effect on the elongation rate constant, various concentrations of JB6 were added to a solution with 50% seeds^37^, before adding tau monomers. In this highly seeded regime, the aggregation is dominated by elongation (see SI, Fig. S3 for a fit with only elongation) and will not change much if k_n_ or k_2_ are reduced (SI, Fig. S4). As seen in Fig. 3D, varying k_+_ describes the data fairly well, indicating that JB6 also has an effect on the elongation rate constant. However, as evident from Fig. 3B, the decrease in k_n_ is many orders of magnitude larger than the decrease in k_+_, with increasing JB6 concentration. The first part of the 50% seeded reaction is further analyzed using linear regression (SI, Fig. S5), yielding a similar reduction in k_+_, compared in Fig. S6.

Since the saturation concentration for k_2_ is $\sqrt{{{{{\rm{k}}}}}_{{{{\rm{M}}}}}}\approx 15$ nM, and aggregation kinetics are not accessible within a reasonable time frame at such low concentrations, it is difficult to quantify the effect on k_2_. We will return to JB6’s effect on k_2_ later when analyzing its binding to fibril surfaces.

Figure 3E shows a comparison of the curve shapes of the last 50% of the kinetic traces, for the non-seeded and 50 % seeded reactions, with 0.1 μM JB6 (see SI, Fig. S7 for 0.01 and 1.0 μM). Intriguingly, the slope is much higher for the non-seeded data. This suggests that JB6 is no longer available for inhibition after the lag phase, in contrast to when JB6 is added to 50% pre-formed fibrils.

### Ultrastructures of endstate aggregates

**Fig. 4:**
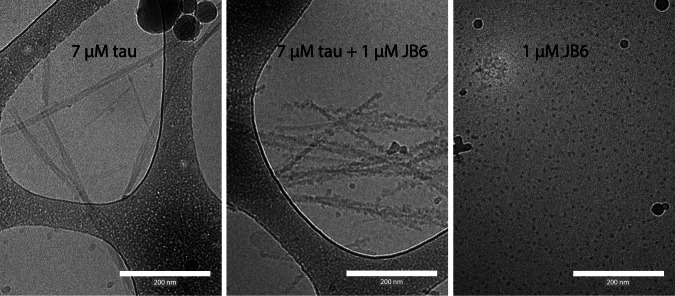
Ultrastructures of endstate aggregates, assessed with cryo-EM imaging. Fibrils formed by 7 μM tau alone (left), aggregates formed in solution with both 7 μM tau and 1 μM JB6 (middle), and of 1 μM JB6 alone (right).

Tau and JB6 were imaged separately, as well as after aggregation in the presence or absence of JB6, using cryo-EM (Fig. 4). The fibrils formed in the presence of JB6 have a granular appearance, suggesting that the aggregates contain both JB6 and tau.

### Depletion of tau and JB6 from solution

**Fig. 5:**
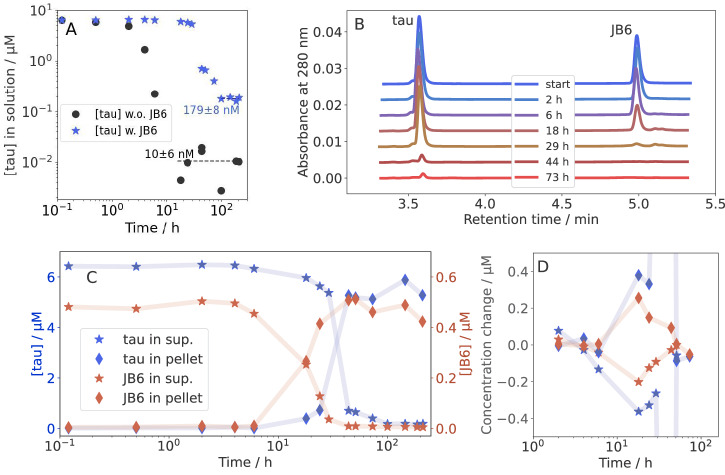
The concentrations of tau and JB6 during an ongoing amyloid fibril formation process, monitored using UV-absorbance in reversed phase HPLC. Prior to measurements, fibrils were pelleted with centrifugation. **A** The concentration of tau in solution. The mean values and standard deviations of the last 7 and 4 points, for tau alone and tau with JB6, respectively, are indicated with text and lines. **B** Chromatograms monitored by the absorbance at 280 nm of the supernatant from a sample with 7 μM tau and 0.7 μM JB6. **C** Concentrations of tau and JB6 in the supernatant and pellet. The concentration in the pellet can be directly compared with the supernatant since pellets were dissolved in the initial volume. **D** The change in concentration between each time point, for both tau and JB6 in supernatant and pellet, with legend shown in (**C**). The purpose of the analysis is to analyze the stoichiometry of tau and JB6 in the pelletable aggregates as a function of time, which is why the y-axis is cropped at  ± 0.45 μM. The value at 44 h for tau is close to ± 5 μM and far outside of the range shown.

Next, we analyze amyloid formation by measuring how the concentration of both tau and JB6 in solution changes during an ongoing aggregation process, using centrifugation and HPLC (Fig. 5). The centrifugation effectively separates the large aggregates from solution (Fig. S8), and HPLC with reversed-phase chromatography is used with UV absorbance detection to quantify the protein concentrations in both the supernatant and dissolved pellet. The aggregation process is accelerated by a magnetic stir bar to minimize chemical modifications of the peptides^35^.

For tau alone, starting from a supersaturated monomer concentration of 7 μM, the monomers in solution decrease in a sigmoidal fashion as tau forms fibrils and reach a plateau of 10 ± 6 nM. In the presence of 0.7 μM JB6, the lag phase is prolonged and the monomer plateau shifts to 179 ± 8 nM (Fig. 5A). As seen in the chromatograms (Fig.5B), tau is essentially kept in solution while JB6 is still in solution, but between 29 and 44 h, JB6 is completely depleted from solution (i.e. the JB6 concentration is below the limit of detection, which is approximately 1 nM), and most of the tau peptide has aggregated. Supporting data in Figs. S9, 10 and Supplementary Note 1 show that a sample of JB6 alone is mostly intact and in solution at this time point, inferring that JB6 is indeed aggregating together with tau in the mixed system. A similar analysis was done at pH 8.0, with no added salt, to investigate the robustness of these findings at a different solution condition, with similar results (Fig. S11). The concentrations of tau and JB6 were calculated from the integrated absorbance of the chromatogram peaks and are plotted in Fig. 5C.

Each pellet was dissolved in the same volume as before supernatant removal and analyzed in the same way (Fig. 5C), enabling direct comparison of the concentrations in the supernatant and pellet. As expected, the amounts in the pellet and supernatant mirror each other. The total JB6 concentration sums up to about 0.5 μM, and the missing 0.2 μM can be ascribed to losses at tube surfaces (Supplementary Note 2). To estimate the stoichiometry of tau:JB6 in the pelletable co-aggregates early in the aggregation process, the change in concentration between each time point is utilized (Fig.5D). This can be seen as a numerical derivative of the data in panel C. In both the supernatant and the pellet, between 18 and 29 h, this stoichiometry is about 2:1 tau:JB6. One should remember that this experiment only detects the pelletable aggregates, and hence, co-aggregates in solution with a different stoichiometry may also form.

### Binding of JB6 to tau fibrils

**Fig. 6:**
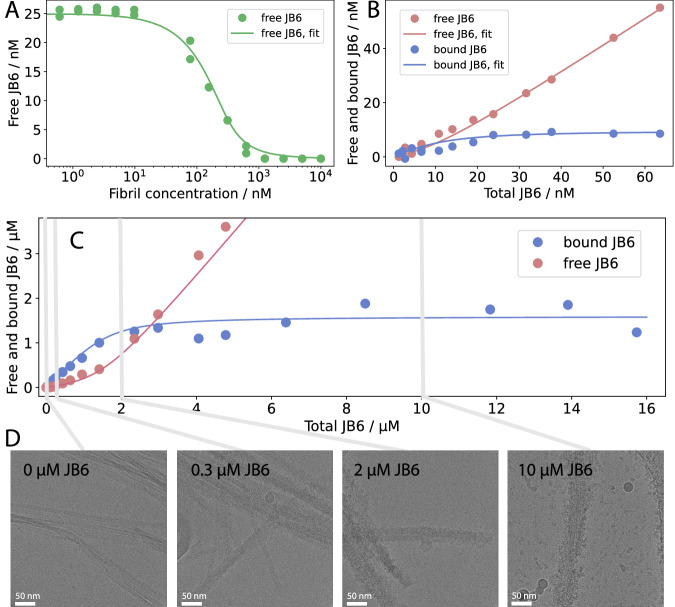
Binding of JB6 to tau fibrils. The free JB6 concentration after incubating JB6 with pre-formed tau fibrils was measured to obtain binding curves. **A** Data from experiments with monomeric JB6 at constant concentration (25 nM) and fibrils at varying concentration. **B** Data from experiments with monomeric JB6 at varying concentration and fibrils at constant concentration (100 nM). The bound JB6 is given by the difference between total and free JB6. The solid lines in (**A**) and (**B**) correspond to a global fit to the data of a standard binding equation, given in SI (Equation 2 and 3), with *K*_*D*_ = 4.7 nM (95% confidence interval 1.6–7.8 nM) and the stoichiometry at saturation, *d* = 0.10 (95% confidence interval 0.08–0.12). **C** JB6 (above its cmc) added to 1 μM pre-formed tau fibrils. The bound JB6 is given by the difference between total and free JB6. The complete data set is given in SI (Fig. S13). The data are fitted to obtain *K*_*D*_ and the stoichiometry at saturation. See Fig. S13 and Supplementary Note 3 for further information on the fits. The fitted curves correspond to *K*_*D*_ = 0.2 μM and *d* = 1.6. Note that this affinity mostly describes micelle binding to fibrils, in contrast to monomeric JB6 in (**A**) and (**B**). **D** Cryo-EM images of four different JB6 concentrations to pre-formed tau fibrils. Tau only (0 μM JB6), below saturation (0.3 μM), at saturation (2 μM), and at large excess (10 μM).

A crucial aspect of JB6 chaperone function is its affinity for fibrils. Since JB6 self-assembles in a micellar-like manner^26^, with monomers coexisting with the micelles^25^, binding must be analyzed with its assembly state in mind. We therefore analyzed fibril binding of both monomeric JB6 (below its cmc, Fig. 6A, B) and micellar JB6 (above its cmc, Fig. 6C, D) by measuring the free JB6 concentration after incubation with pre-formed fibrils. The fibril-affinity of monomeric JB6 was determined using two data sets: one with varying fibril concentration and constant JB6 concentration of 25 nM (panel A), and one with varying JB6 concentration and a constant fibril concentration of 100 nM (panel B). A global fit to both data sets was performed using a standard equation for independent binding (Equations 2 and 3 in the SI), yielding a *K*_*D*_ of 4.7 nM (95% confidence interval 1.6–7.8 nM) and a stoichiometry at saturation, *d*, of 0.10 (95% confidence interval 0.08–0.12), corresponding to 1 JB6 molecule per 10 tau molecules in the fibril state.

At JB6 concentrations above its cmc, the distribution between bound and free JB6 will depend on the fibril-affinity of both monomers and micelles. The free JB6 concentrations after binding to 1 μM fibrils are shown in Fig. 6C. The bound JB6 is calculated as total minus free. Fits resulted in *K*_*D*_  ≈ 0.2 μM and *d*  ≈ 1.6. This affinity should be seen as an upper limit of the *K*_*D*_, i.e., a lower limit of the affinity, since JB6’s self-assembly is concentration dependent and different assembly states likely provide different binding stoichiometries and affinities to tau fibrils. See SI, Fig. S13 and Supplementary Note 3, for detailed fitting of this data set, including an adsorption isotherm of JB6.

Although affinity analysis is less straightforward above the cmc, higher concentrations enable complementary cryo-EM analysis. Figure 6D shows cryo-EM images at different JB6 concentrations and 1 μM fibrils. At 0.3 μM JB6, fibrils appear more granular and no free JB6 is observed. At 2 μM JB6, the fibrils are saturated with JB6 and almost no free JB6 is observed. At 10 μM JB6, well above saturation, highly coated fibrils are seen to coexist with free JB6 micelles that are not associated to any fibril. The increased thickness upon fibril coating supports the finding that about 1.6 JB6 molecules can bind per tau monomer in the fibril state. The coating of the fibril surface by JB6 may be associated with an effect on secondary nucleation.

### Lack of binding of monomeric tau to JB6

**Fig. 7:**
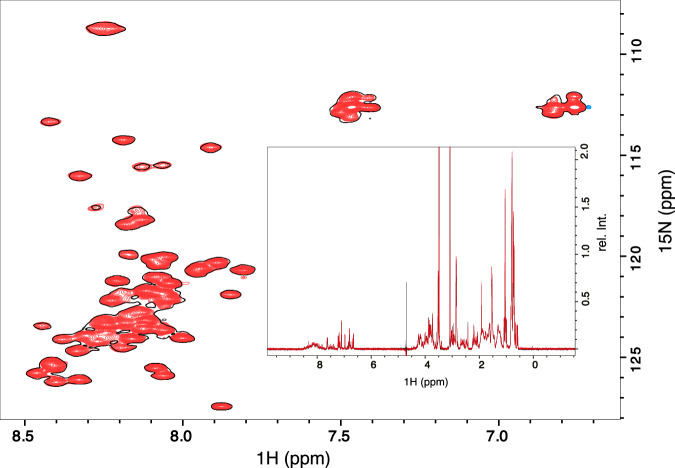
NMR spectra of monomeric tau in presence and absence of JB6. ^1^H-^15^N-2D and ^1^H-1D NMR spectra on ^15^N-labeled tau in red and 1:1 molar ratio of tau + non-labeled JB6 in black contour lines. The 2D-spectra are shown using the same contour levels, with 40 levels plotted of tau alone, and only the lowest level of the tau + JB6 sample, to allow for comparison. The inset of the 1D spectra shows the complete water suppressed ^1^H spectrum of the same samples, referenced to DSS. Copies of all spectra are shown in large scale as Supplemental Data.

To examine whether JB6 interacts with tau monomers, we first performed ^1^H-^15^N-2D and ^1^H-1D NMR experiments on tau labeled with ^15^N, followed by the same experiments with the addition of natural abundance JB6 to the NMR tube and gentle mixing in a 1:1 molar ratio (Fig. 7). To analyze the potential interaction with JB6 in a similar solution condition as the aggregation kinetics and solubility measurements, a 20 mM NaP buffer at pH 7.4 with 100 mM NaCl was used at 20 ^∘^C (the signal was too weak at 37 ^∘^C for reliable measurements). The 2D NMR experiment shows correlations between the N-H amide bonds of tau, thus providing atomic-resolution information primarily on the backbone amides, but also on the side-chain amides of the three glutamines and three asparagines. Any structural change in the tau peptide chain due to interaction with JB6 would be observed as peak shifts or changes in intensity. The narrow dispersion of the tau backbone amide peaks, especially in the ^1^H-dimension with a spread of less than 1 ppm (7.75–8.5 ppm) is a clear sign that tau is mostly disordered. The two clusters of peaks at (7.5; 113) and (6.8; 113) ppm represent the 6 side chain amides. A change due to side-chain interaction of tau with JB6 would also have been seen by studying the 1D NMR spectral profile of the respective sample (the inset in Fig. 7), especially the methyl peak region (0.5–0.9 ppm), the aliphatic region (0.5–2 ppm) and the aromatic side chain region around 7 ppm. Due to dilution when adding JB6, the data corresponding to the mix of the two proteins (black color) has been multiplied with a factor 9/8 before plotting. In conclusion, neither the peak intensity nor the chemical shift of the 2D or 1D peaks change notably upon addition of JB6, thus no direct interaction between the two proteins could be detected.

## Discussion

The interplay between JB6 and tau has so far been studied in cells, where Chang et al.^7^ and Esquivel et al.^6^ reported that overexpression of JB6 reduces tau aggregation, whereas down-regulation has the opposite effect. In this work, we aim to understand these observations in a pure system in order to dissect the kinetic and thermodynamic effects of JB6 on tau aggregation. The current results reveal a very high potency of JB6 as a suppressor of tau aggregation; this is observed both as a retardation of fibril formation at very low sub-stoichiometric ratios of JB6:tau and as an increase in tau solubility. These in vitro results show a clear effect on protein level and are important given the close link between tau aggregation and pathologies, such as AD and multiple primary tauopathies.

The remarkably low substoichiometric molar ratio, with as little as 1:700 JB6:tau producing a three-fold increase in lag time (Fig. 2A), cannot be explained by interactions between JB6 and tau monomers alone, and therefore implies that JB6 interacts with rare species, such as fibril defects^38^ or transient species in solution, including oligomers^39^. In the present work, we set out to identify the mechanistic origin of the potent inhibition, i.e., resolve the effect of JB6 over discrete microscopic steps in the tau aggregation process. To this end, we used the amyloid-prone core fragment of tau in a buffer system devoid of additives. Importantly, the data show that JB6 is a potent inhibitor both when the process starts from pure tau monomers and when it starts from monomers in the presence of 1% pre-formed tau fibrils, which otherwise strongly accelerate aggregation. These observations imply that JB6 interferes with both primary and secondary pathways in the amyloid fibril formation. We note steeper transitions for the non-seeded reactions with 10 nM JB6 compared to at 1% seeds (Fig. 2E), and we cannot exclude the possibility that co-aggregates that form in the presence of JB6 have a morphology with slower secondary processes.

To provide more detailed insights and to quantify the level of suppression, we fitted a model, implemented in the AmyloFit platform^36^, “multistep secondary nucleation dominated”, which has previously been shown to describe tau fibril formation well^30^. Consistent with this report, the monomer concentration at half saturation of secondary nucleation, $\sqrt{{{{{\rm{k}}}}}_{{{{\rm{M}}}}}}$, is low (tens of nM). The rate constant for primary nucleation, k_n_, is also low compared to other AD-related peptides, such as A*β*42 and A*β*40^40^, whereas the rate constant for elongation, k_+_ is comparable and k_2_ high (but saturated).

To dissect the effect of JB6 on these microscopic steps, we monitored the aggregation kinetics starting from monomers supplemented with seeds at different concentrations. Without seeds, fibril formation depends strongly on primary nucleation. Under these conditions, the data are well explained by changes in k_n_, indicating that JB6 strongly suppresses primary nucleation, as is also observed for other amyloid peptides^12,16,41^. With 50% seeds, growth is dominated by elongation, and even here, JB6 slows down fibril formation, showing that k_+_ is reduced as well, but to a much smaller extent than k_n_. With such fast aggregation, we stress the importance of pre-equilibrating JB6 at as close to the final concentration as possible, to allow dissociation of JB6 into its active subunits^17^, before the addition of tau. The dissociation of JB6 into monomers occurs on the time scale of one hour at 37 °C^25^, below its critical micelle concentration of about 120 nM^26^. Since the saturation concentration for secondary nucleation of tau is very low, tens of nM, aggregation kinetics alone can not be used to investigate whether JB6 targets k_2_. However, cryo-EM images in Fig. 6 show that JB6 binds to fibril surfaces and completely coats the fibrils, which strongly suggests that JB6 also affects k_2_.

The remarkable potency of JB6 at sub-stoichiometric ratios to tau is paralleled by its effects on amyloid-*β*^12,16^, *α*-synuclein^21^, and polyglutamine peptides^41^. Comparison of data from non-seeded and 1% seeded samples (Fig. 2) provides insight into the stoichiometry of JB6 action. The effect of seeding with 1% (70 nM) tau fibrils is essentially abolished by 100 nM or 1.0 μM JB6, but not by 10 nM JB6. The stoichiometry for complete inhibition is thus between 7:1 and 7:10 of aggregated tau: JB6. In contrast, for reactions starting from 7 μM tau monomers alone, as little as 10 nM JB6 is sufficient to delay the process through an inhibition of primary nucleation. This is consistent with JB6 acting on oligomeric tau species that are intermediates in the aggregation process, i.e., nucleation is hindered by the formation of co-oligomers between chaperone and client.

NMR spectroscopy (Fig. 7) fails to detect any interaction between tau monomers and JB6, whereas the binding assay (Fig. 6A, B) shows that JB6 binds to mature tau fibrils with high affinity, *K*_*D*_  ≈ 5 nM.

The depletion experiments, followed by HPLC, are started from monomeric tau and JB6. Here, we find a high content of JB6 in the early aggregates (Fig. 5D). One plausible interpretation of these results is that once all JB6 is adhered to aggregates, thus depleted from solution, newly formed nuclei of tau may rapidly convert into elongation-prone structures, which is seen as a fast aggregation process. Another explanation is that, after all, JB6 is depleted from the solution into co-oligomers of a certain JB6:tau ratio, the co-oligomers gain more tau, and eventually the JB6:tau ratio allows for nucleation of species with fast elongation. Both options would explain the steep aggregation slope observed after a prolonged lag phase (Fig. 3E).

Using HPLC to quantify the contents of the pellet and supernatant during fibril aggregation and suppression provides a direct measure of aggregate mass, which complements the indirect measurement using a fluorescence dye, such as X34. Thus, the depletion experiment confirms that JB6 prolongs the lag phase of tau aggregation (Fig. 5A). In this experiment, we also observe an increase in apparent tau solubility when JB6 is present during fibril formation. Similar findings have been reported for A*β*42^15^ and *α*-synuclein^21^.

A plausible explanation for the increase in apparent solubility is that JB6 has a very high chemical potential on its own. If, upon co-assembly with an amyloid protein, JB6 lowers its own free energy substantially, this may allow the free energy of the amyloid to increase, yet with a net-negative change in the total free energy of the system. Consequently, a system consisting of co-assemblies and a relatively high concentration of free amyloid protein can have a lower free energy than the system consisting of pure amyloid aggregates, free chaperone and a lower free amyloid protein concentration. The latter, therefore, does not represent an equilibrium state. Such a mechanism may result in a higher solubility of the amyloid peptide in the presence of JB6 compared to the peptide alone^24^.

The ultrastructures seen with cryo-EM imaging (Fig. 4) support that JB6 ends up with the aggregates when fibrils are formed in the presence of JB6. This may either correspond to coating of fibrils after they have formed (as examined in Fig. 6) or JB6 may induce—and become incorporated in—another fibril structure. To distinguish between these scenarios, we can compare the concentration of free JB6 at the end-point with the concentration of free JB6 that would exist after addition to pre-formed fibrils at the same total composition, based on the measured affinity and stoichiometry from Fig. 6A, B, using Equation 2 and 3 in SI. This tells us that JB6, in the case of coating of pre-formed fibrils, should be present to a measurable extent (20 nM, see Supplementary Note 4 for details, which is well above the detection limit of about 1 nM). However, there is no observable JB6 peak in the chromatogram after tau aggregation together with JB6, indicating that the free JB6 is below 1 nM and thus more likely incorporated in a co-aggregate with tau, which represents a different end-state. Further findings indicating that the presence of JB6 promotes a new kind of aggregate, are the increased tau concentration after aggregation (Fig. 5A), and the observation that non-seeded aggregation has a steeper growth after t_1/2_ compared to when JB6 is added to 50% seeds (Fig. 3E and S7). If the JB6 that is present from the start of the aggregation only coats the formed fibrils, the growth after 50% fibril mass should look the same as when JB6 is added to 50% seeds, which is clearly not the case. These findings, together, indicate that JB6-coating of fibrils is not the same end-state as the one that forms when JB6 is present from the start of aggregation. Determining the structural details of this interaction and the formed co-aggregates will be the focus of future studies.

Here, we demonstrate the potency of JB6 in inhibiting tau amyloid formation by acting on multiple microscopic steps: primary nucleation, secondary nucleation and elongation. We also show that JB6 binds to tau fibrils with a high affinity, and when present during the fibril process, it may increase the solubility of tau by co-aggregating together with tau. This multifaceted mechanism of action, which alters kinetic and thermodynamic parameters, may form the basis for the development of a new class of therapeutics.

## Methods

All chemicals were of analytical grade. All buffers were filtered through a wwPTFE-filter with 0.22 μm pore size and degassed.

### Preparation of JB6

Expression and purification of JB6 with the sequence, MVDYYEVLGVQRHASPEDIKKAYRKLALKWHPDKNPENKEEAERKFKQVAEAYEVLSDAKKRDIYDKYGKEGLNGGGGGGSHFDSPFEFGFTFRNPDDVFREFFGGRDPFSFDFFEDPFEDFFGNRRGPRGSRSRGTGSFFSAFSGFPSFGSGFSSFDTGFTSFGSLGHGGLTSFSSTSFGGSGMGNFKSISTSTKMVNGRKITTKRIVENGQERVEVEEDGQLKSLTINGKEQLLRLDNK, was done as previously described in^42^. Concentration determination was obtained using absorbance spectroscopy, with an extinction coefficient at 280 nm of 14,440 cm^−1^ M^−1^, using a Labbot instrument. The protein was flash-frozen using pre-cooled metal blocks and tubes (−80 °C) after purification, then stored at −20 °C until thawing prior to use. The purity of purified JB6 was assessed with SDS PAGE and analytical HPLC, providing at least 99.6 % purity level, as shown in Fig. S14.

### Preparation of tau

The AD amyloidogenic core of Tau, 304-380C322S (numbering according to 441 2N4R tau) with the sequence: MGSVQIVYKPVDLSKVTSKSGSLGNIHHKPGGGQVEVKSEKLDFKDRVQSKIGSLDNITHVPGGGNKKIETHKLTFRE was transformed, expressed and purified as previously described in detail in ref. ^30^. Note that the initial M is cleaved off during translation by E. coli, and is not present in the final purified protein. To summarize the purification, codons were optimized for E. coli to obtain better expression and the sequence was inserted in a Pet3a plasmid, purchased from Genscript, USA. The plasmid was transformed in E. coli (BL21 Star DE3 pLysS) from Invitrogen, USA. Overexpression was initiated through autoinduction with overnight express medium. The labeling of tau with ^15^N- and ^3^H was achieved through expression in M9 minimal medium containing ^15^NH_4_Cl as the sole nitrogen source, or supplemented with tritiated D-glucose (PerkinElmer) 5 minutes before induction. Protein expression was induced using isopropyl *β*-D-1-thiogalactopyranoside (IPTG). Detailed protocol for media recipe can be found in ref. ^43^.

After harvesting of the cells by centrifugation, they were resuspended and sonicated to break open the cells. The lysed cells were again centrifuged, and the soluble fraction was boiled to denature endogenous soluble E. coli proteins, which were removed by centrifugation. The remaining fraction was passed through a Q Sepharose resin to get rid of any negatively charged species, after which two steps of SPHP followed, first at pH 8.0, then at pH 7.0, where the protein was eluted with a salt gradient. Subsequently, the pure tau monomers were subjected to SEC on a 26 × 600 mm Superdex75, before being aliquoted, freeze-dried and stored at −20 ^∘^C until further use. For the detailed protocol, we refer to ref. ^30^. The sample purity was examined using SDS PAGE, mass spectrometry, absorbance measurements, and HPLC, providing  ~ 99.5% purity level, as showed in Figu. S15. To ensure high homogeneity of tau monomers prior to each experiment, the protein was always subjected to a final SEC right before usage. A freeze-dried aliquot of tau monomers was reconstituted in 6 M guanidine hydrochloride and injected on a Superdex75 10/300 column, pre-equilibrated in degassed and filtered experimental buffer. The final protein concentration was determined by calculating the integral of the chromatogram with 280 nm absorbance and an extinction coefficient of 1490 M^−1^ cm^−1^. The freshly isolated monomers were always kept on ice until the start of the experiment.

### Aggregation kinetics

Freshly isolated tau monomers were mixed with X34, and 1% seeds if applicable, and added to a PEGylated low-binding 96-well plate, half area (3881, Corning), with a multichannel pipette just before measuring the fluorescence in a plate reader (FLUOstar Omega, BMG LABTECH) at 37 ^∘^C. The buffer was 20 mM NaP, pH 7.4, 0.2 mM EDTA, 150 mM NaCl. The seeds were prepared in the same buffer, with the same concentration of X34 as used in the aggregation kinetics (2 μM), with 14 μM tau, and a magnetic stirring bar (500 rpm) at 37 °C overnight. The seeds were vortexed briefly to homogenize the dispersion prior to the aggregation kinetics assay, to obtain an accurate seed concentration. The final concentrations of all components were 7 μM tau monomers, 0.07 μM tau fibrils in the 1% seeding case, 2 μM X34 (based on findings in ref. ^30^, and JB6 at 0, 0.01, 0.1, and 1 μM. Since JB6 needs to dissociate into monomers to be fully active as an inhibitor^17^, and this dissociation occurs on the timescale of about 1 h at 37 °C^25^, JB6 was pre-diluted to twice the final concentration directly in the plate, at half the final volume. The plate was incubated with lid for 2 h at 37 °C before monomeric tau was added and the reaction started. The total volume in each well was 100 μl, with five wells of each condition. The plate was sealed with a plastic cover to prevent evaporation. Continuous reading with excitation of 380 nm and emission at 480 nm was done to probe the X34 fluorescence.

The aggregation kinetics at 50% seeds were performed in the same way, with the following few exceptions. Since the aggregation happens on the timescale of minutes, the seeds were first added to the pre-equilibrated JB6 and left to incubate together for two minutes, giving JB6 a chance to interact with the seeds before the tau monomers were added. The number of replicates was restricted to three, at JB6 concentrations of 0, 0.003, 0.01, 0.03, 0.1, 0.3, 1 μM, and the addition of the tau monomers was performed with a multichannel pipette (briefly mixing up and down) to make it possible to load all wells and start reading within 1 minute. The final tau concentrations were 3.3 μM monomers and 3.3 μM of pre-formed fibrils, which were grown at the same buffer conditions with a magnetic stirring bar (500 rpm) at 37 °C.

### Depletion of tau and JB6 from solution: centrifugation and HPLC

Samples with either 7 μM monomeric tau, or 7 μM monomeric tau together with 0.7 μM JB6, were incubated at 37 °C in a 5 ml Eppendorf protein low-binding tubes, in an initial volume of 5 ml, with a magnetic stirring bar at 500 rpm. 200 μl was removed to a 1.5 ml Eppendorf protein low-binding tube at the following times after mixing: 0, 0.5, 2, 4, 6, 18, 24, 29, 44, 51, 73, 100, 155, 187, 211 h. At each timepoint, the sample was centrifuged in an Eppendorf Centrifuge 5424 at 20,000 g for 30 min. 100 μl of the supernatant closest to the surface was removed and analyzed using HPLC (Shimadzu Nexera X3) in the follownig way: 40 μl of the sample was loaded on a 60 °C C18 reversed phase column (BIOshell A160 Peptide CN column 66966-U, Sigma-Aldrich). Elution was done with a 10 min linear gradient of 5–95% acetonitrile in an aqueous mobile phase, with 0.1% TFA, at 0.5 ml/min. Absorbance at both 205 and 280 nm were measured and used to quantify the protein concentrations. The rest of the supernatant was discarded and the pellet dissolved in 8 M urea to the same volume as before removal of supernatant, then analyzed with HPLC and SDS PAGE. In the SDS PAGE, the sample was premixed with a loading buffer with 10% SDS (final concentration protein was 5/3 times the original sample concentration) and heated to 80 °C for 2 min, before, 10 μl were loaded in each lane. The electrophoresis was run for 45 min at 120 V). The plate used for the HPLC auto-sampler (kept at 10 °C) was a PEGylated low-binding 96-well plate, half area (3686, Corning). Careful consideration of the vessels used was taken to reduce the adsorbed amount in each step. To estimate the protein losses at surfaces in the measurement process (centrifugation and HPLC), different concentrations of both JB6 and freshly isolated tau monomers were measured with HPLC both directly after sample preparation and after centrifugation and incubation in the HPLC sample plate for 2 h (Fig. S12). The surface activity of JB6 has been studied before^44^. To evaluate concentrations in the nanomolar range of tau, a standard curve was run with the same method in the HPLC. The linear regression was weighted with concentration^−1^ (Fig. S16). The buffer used was 20 mM NaP, 0.2 mM EDTA, pH 7.4, 150 mM NaCl.

### Affinity determination of JB6 towards tau fibrils

JB6 was serially diluted to obtain concentrations at twice the final concentration, in 100 μl (in protein low-binding tubes, Eppendorf). The samples were pre-incubated for 3 h at 37 °C to reach its equilibrium size distribution. 10 μl of each sample was run on HPLC, with settings given in the previous section, for measuring the actual concentration of JB6 in solution before 90 μl of 2 μM tau fibrils were added (Fig. S13). The fibrils had been let to grow from monomers during 70 h, with a magnetic stirring bar (500 rpm) at 37 °C. The buffer used was 20 mM NaP, 0.2 mM EDTA, 150 mM NaCl, pH 7.4. After the addition of fibrils to JB6, the samples were let to incubate overnight (17 h) at 37 °C, followed by centrifugation in the same tubes at 20,000 g for 30 min. The concentration JB6 in the supernatant was quantified using HPLC, obtaining the free JB6 concentration.

### Cryo-EM imaging

Tau fibrils were formed as described in “Affinity determination of JB6 towards tau fibrils” and JB6 was added in concentrations of 0, 0.3, 2, and 10 μM. After overnight incubation at 37 °C, specimens for cryo-TEM were prepared in an automatic plunge freezer system (Leica EM GP). The climate chamber temperature was kept at 21 °C, and relative humidity was ≥90% to minimize loss of solution during sample preparation. 4 μL sample was loaded onto 300 mesh lacey carbon-filmed copper TEM grids and blotted with a filter paper to absorb extra solution. The grids were then plunged into liquid ethane (−180 ^∘^C) to flash freeze all samples, and stored in liquid nitrogen until imaging. Imaging was performed on a Thermo Fisher Glacios 2 Cryo-TEM equipped with Falcon 4i direct electron detector with FFI and AFIS imaging and an acceleration voltage of 200 kV.

### NMR spectroscopy

All NMR spectra were recorded using a Bruker Avance Neo 800 MHz spectrometer with a TCI CryoProbe (Bruker Biospin, Rheinstetten, Germany), operating at a ^1^H resonance frequency of 800.14 MHz. Besides the specified addition of tau and JB6, the NMR samples were prepared with 10% D_2_O, and minute amounts of chemical shift reference DSS (Sodium 3-(trimethylsilyl)propane-1-sulfonate) in a volume of 550–600 μL in a 5 mm tube. The 1D ^1^H spectra were recorded using water suppression at 4.7 ppm with excitation sculpting^45^ combined with a perfect echo^46^ using the standard Bruker pulse-program zgesgppe. The spectral width was set to 15.6 ppm with 32,768 points and an acquisition time of 1.3 s. 128 scans were recorded with an interscan delay of 1.0 s, giving a total experimental time of 5 min. 2D ^1^H-^15^N SOFAST heteronuclear multiple-quantum coherence (HMQC) was performed using the standard Bruker pulse-program sfhmqcf3gpph^47^, with 52 scans and 128 points in the indirect dimension over a spectral width of 26 ppm, an interscan delay of 0.2 s and an acquisition time of 40 ms, giving a total experiment time of 30 minutes. The experiments were carried out at 20 °C and the spectra were processed with TopSpin 4.3 using zero filling, linear prediction and cosine squared window function for the 2D-data sets, and exponential window multiplication of the FID with line broadening 0.3 for the 1D data sets.

The sample with 18 μM tau was analyzed first. Next the JB6 was added to final concentrations of tau and JB6 of 16 μM. The intensities of the 1D-spectra from the sample with both tau and JB6 are scaled with a factor 9/8 to account for the dilution of tau, allowing for direct comparisons between the intensities from the two data sets.

### Reporting summary

Further information on research design is available in the Nature Portfolio Reporting Summary linked to this article.

## Supplementary information

Supplemental information

Description of Additional Supplementary Files

Supplemental data

Reporting Summary

## Data Availability

The data used to generate the figures in this study have been deposited in https://github.com/saralinse/Published_Data/tree/CommsChem2026-JB6-tau. The data include aggregation kinetic traces, calculated lagtimes and rate constants, calculated peak integrals of HPLC chromatograms, and NMR data.
