# Supplementary material for: Human chaperone DNAJB6b suppresses tau fibril formation through co-aggregation
Source: Commun Chem. 2026 Aug 1;9:263. doi: 10.1038/s42004-026-02108-1 (PMC13434469; doi:10.1038/s42004-026-02108-1)
Supplement: Supplementary file 1 [file 42004_2026_2108_MOESM1_ESM.pdf]

Supplementary Information to

Human chaperone DNAJB6b suppresses tau fibril formation through co-aggregation

Andreas Carlsson[1] †*, Emil Axell[1] †*, Johan Wallerstein[1], Dev Thacker[1], Ulf Olsson[2], Sara Linse[1]

†These authors contributed equally
*Corresponding authors. Email: andreas.carlsson@chem.lu.se, emil.axell@chem.lu.se

This PDF file includes:

Supplementary Notes 1-4
Figures S1-S16

Supplementary Note 1: Concentration of soluble JB6 as a function of time

To understand how JB6 behaves without tau present, a sample with only 700 nM JB6 was analyzed in parallel with the samples discussed in Figure 5. The buffer, sample volume, stirring, and temperature were the same, and withdrawals were subjected to the same centrifugation process and quantification with HPLC, UV absorption, as shown in Figure S9 together with the supernatant concentrations of tau and JB6 from Figure 5. Some JB6 is pelletable at longer times and about half of it is left in solution at the end of the experiment, after 211 h. The decrease of soluble JB6 is probably due to aggregation induced by chemical modifications, likely enhanced by the stirring, since the unmodified JB6 forms micelles that are stable in size [1]. However, at the point where no JB6 is left in solution in the sample with tau present, about after 30 h, still about 80 % of the initial JB6 is left in solution. Hence, the dramatic decrease of JB6 in the presence of aggregating tau cannot be ascribed to JB6 self-aggregation. The results are supported by SDS PAGEs of the pellets of the same samples, Figure S10.

To find out how much centrifugation is needed to sediment tau fibrils at the solution condition of 20 mM NaP buffer, 0.2 mM EDTA, pH 7.4, and 150 mM NaCl, different centrifugation times at 20 000 g was used and the concentration tau in the supernatant was quantified with HPLC and ThT fluorescence (Figure S8). Since the amount of sedimentation is also dependent on the distance the particle travel, the same tubes and volumes where used, both in this experiment and in the main article. Samples of 200 µl was centrifuged in 1.5 ml protein low binding Eppendorf tubes and 100 µl was carefully removed from the surface layer. The 7 µM tau sample had been incubated at 37 °C with stirring for 48 h before measurements, at which point no more detectable fibril growth is seen during these conditions. With ThT

fluorescence, we conclude that the fibrils are depleted from solution already after 10 min, which is seen both in the supernatant and the pellet (resuspendend in the same volume). With HPLC UV absorbance at 280 nm, the sensitivity is greater and we can understand that after 20 min the measurements are robust.

Supplementary Note 2: Surface adsorption of tau and JB6 in centrifugation experiments

In Figure S13, the surface adsorption of JB6 in Eppendorf low binding tubes was investigated. Unfortunately, when measuring the concentration of tau and JB6 after centrifugation, as done for the data in Figure 5, both Eppendorf low binding tubes and a waiting time in the HPLC plate (PEGylated 96 well plate, half area) for auto-injection is encountered to the samples. Hence, knowledge of the extent of surface adsorption in this specific set-up is needed, especially when quantifying concentrations in the low nM-range. This was investigated by measuring several concentrations of both proteins, first directly after dilution, with minimal exposure of surfaces, then, after incubation in the same containers and volumes as the samples from Figure 5. These include dilution in a 5 ml Eppendorf protein low binding tube, incubation 1 h, then incubation in a 1,5 ml protein low binding tubes (since the samples were centrifuged for 30 min), and finally incubation in the HPLC plate (PEGylated 96 well plate, half area, volume 100 µl) for 1 h, before measuring. From Figure S12 we conclude that JB6 has more surface adsorption than tau with this methodology. It may be noted that at 700 nM JB6 concentration, about 25 % are adsorbed. This is why the JB6 concentration in Figure 5 starts at 700 nM * 0.75 ≈ 500 nM in the supernatant (and end at this concentration in the dissolved pellet). Tau adsorbs less at these surfaces, but at 10 nM (which is close to the measured solubility of tau alone), about half of the sample is depleted from solution. The time exposed to the various surfaces varied slightly, which explains the large variance of the solubility of tau alone.

Supplementary Note 3: Binding of JB6 to surfaces and tau fibrils

Since JB6 is known to be highly surface active [1, 2], the concentration of JB6 after incubation at twice the final concentration was measured using HPLC, absorbance at 280 nm. This is the actual concentration JB6 in each sample when tau fibrils are added, and is used as the total JB6 concentration since this is the accessible JB6 concentration in solution. As seen in Figure S13A-B, the concentration is similar to the initial concentration for high JB6 concentrations, but at less than a few µM the surface adsorption is obvious. An adsorbed amount can be calculated using the difference in initial and measured concentration after incubation, the sample volume of 100 µl, and the surface area (in Eppendorf protein low binding tubes, size 1.5 ml) which is approximated from inner tube measures according to Eppendorf's technical data sheet, to obtain an area of 1.2 cm^2. Figure S13 shows the outcome of the calculations, with the adsorbed amount, Γ, in mg/m^2. An adsorption isotherm fit is included, given by Equation 1:

$$\Gamma = \frac{\Gamma_{max} * K * [JB6]_{eq}}{K * [JB6]_{eq} + 1} \tag{1}$$

where Γ_{max} is the maximum adsorbed amount, fitted to 41 mg/m^2, $[JB6]_{eq}$ is the JB6 concentration at equilibrium, and K is the equilibrium constant, fitted to $0.24 * 10^6$ M. The affinity is seen as a lower limit due to the concentration dependence of JB6 self-assembly, which likely skew the adsorbed amount to higher values with increasing concentration. The surface adsorption of JB6 to silica has been investigated before [2], with an adsorbed amount of ≈12 mg/m^2, $[JB6]_{eq}$ at 0.75 µM JB6. The discrepancy can be ascribed to concentration dependence, different surface material, and uncertainty in the surface area due to surface roughness.

In Figure 6, the binding of JB6 to tau fibrils are studied by adding 1 µM tau, c_{tau}, to different concentrations JB6, c_{JB6} (measured prior to the addition of tau, as outlined above, with HPLC). In the fitting of the data to Equation 2 and 3, two parameters are fitted, K_D (dissociation equilibrium constant) and d (stoichiometry at saturation).

$$[JB6]_{free} = -0.5 * (K_D + c_{tau} * d - c_{JB6}) + \sqrt{0.25 * (K_D + c_{tau} * d - c_{JB6})^2 + c_{JB6} * K_D} \quad (2)$$

$$[JB6]_{bound} = \frac{c_{tau} * d * [JB6]_{free}}{[JB6]_{free} + K_D} \quad (3)$$

The data span a large interval of concentrations, which is why we need to be careful when fitting. Since d is best determined with the highest concentrations of JB6, this parameter is fitted using linear y-scale (providing greatest weight to the largest values). But, K_D is not best fitted to the largest values, but to all of them with equal weight in the fits. (The K_D obtained with the linear y-axis scale was 460 nM, with a large 95 % confidence interval of 63-856 nM. This ill determined K_D originates from that the values at the highest JB6 concentrations mostly determine the fit.) To fit K_D using more evenly distributed data, which will be more sensitive to changes in K_D, the free JB6 concentration is log10 transformed. With this transformation, d is ill determined and is hence locked to 1.6 as obtained with the fit to linear values. K_D is now fitted to 205 nM, with 95 % confidence interval of 127 to 283 nM.

Supplementary Note 4: Comparison of JB6 binding to fibrils: coating or co-assembling?

As a way of understanding if the presence of JB6 during tau aggregation promotes a different kind of structure compared to when JB6 is added to pre-formed fibrils, the affinity parameters obtained in Figure 6 can be used to calculate a lower limit of non-bound JB6 concentration, given that the binding site is the same. If another concentration is found when fibrils are formed in the presence of JB6, it indicates that the JB6-tau complex is different from the coating situation. The measured concentration after aggregation in the presence of JB6 (Figure 5) is below the detection limit of the method, which is approximately 1 nM. The concentration expected if the binding is the same is at least 11 nM, calculated using Equation 2 and 3, with different JB6 and tau concentrations to check for robustness of the estimation. JB6 is added at 0.7 µM to 7 µM tau monomers. Inserting these numbers in Equations 2 and 3 gives 61 nM free JB6 (monomeric JB6-affinity and stoichiometry) and 13 nM (micelle B6-affinity and stoichiometry). If we instead use the actual measured concentrations of aggregating

tau and JB6 (Figure 5C) — which likely deviates from the initially mixed concentrations due to surface adsorptions as investigated in figure S12) — 6 µM tau and 0.5 µM JB6, the corresponding calculations render 20 and 11 nM free JB6. The most likely value to expect is 20 nM, since this correspond to the measured fibril amount and available JB6 after surface adsorption, with monomeric JB6 since the aggregation happen in the time scale of tens of hours and JB6 micelles dissociate with a half-time of about 20 min at the temperature of question, 37 °C. But also without any assumptions, the lowest value is 11 nM, which is about an order of magnitude above the detection limit. Therefore, this experimental comparison indicates that JB6 does not simply coat formed fibrils when present during aggregation, but another type of tau-JB6 complex is formed together with JB6.

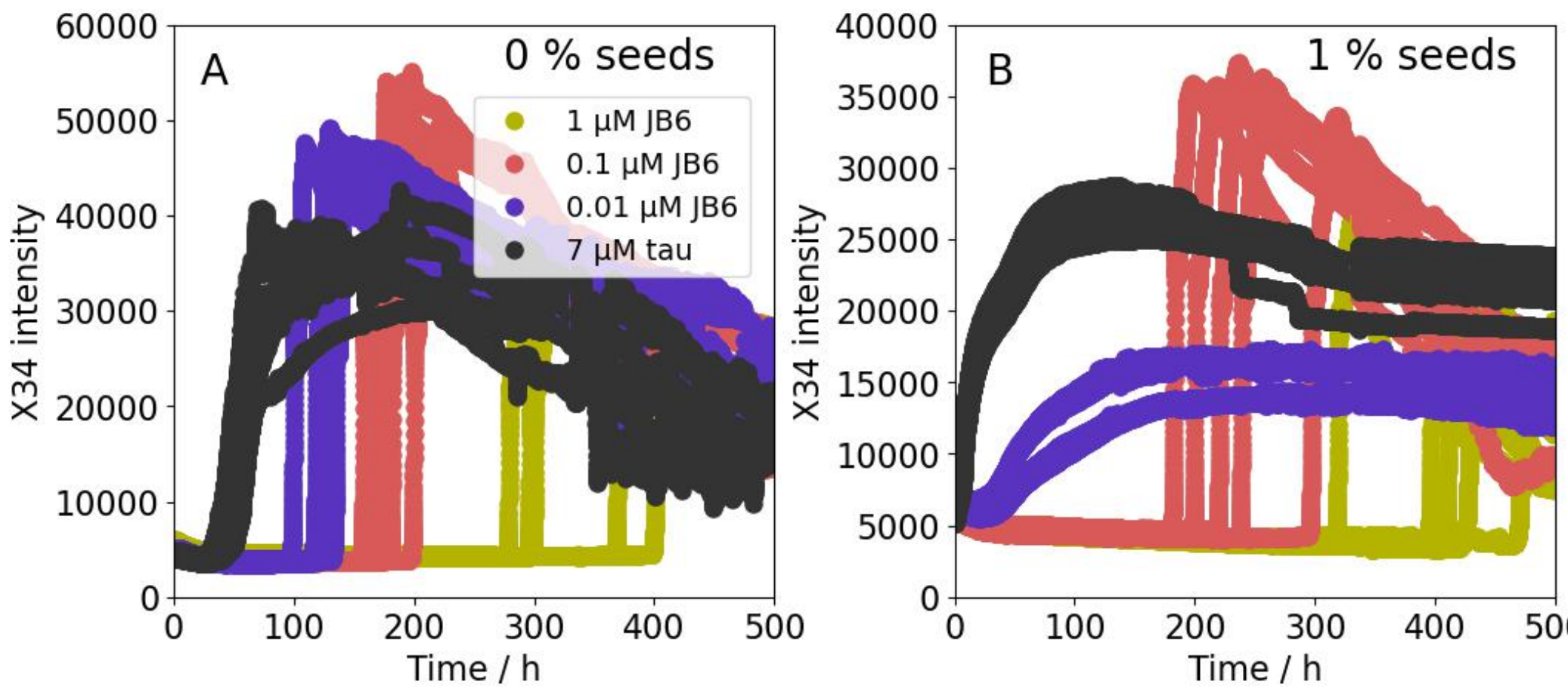

Figure S1: Raw data of the kinetic traces used in Figure 2. **A**: Non-seeded aggregation. **B**: 1 % seeded aggregation.

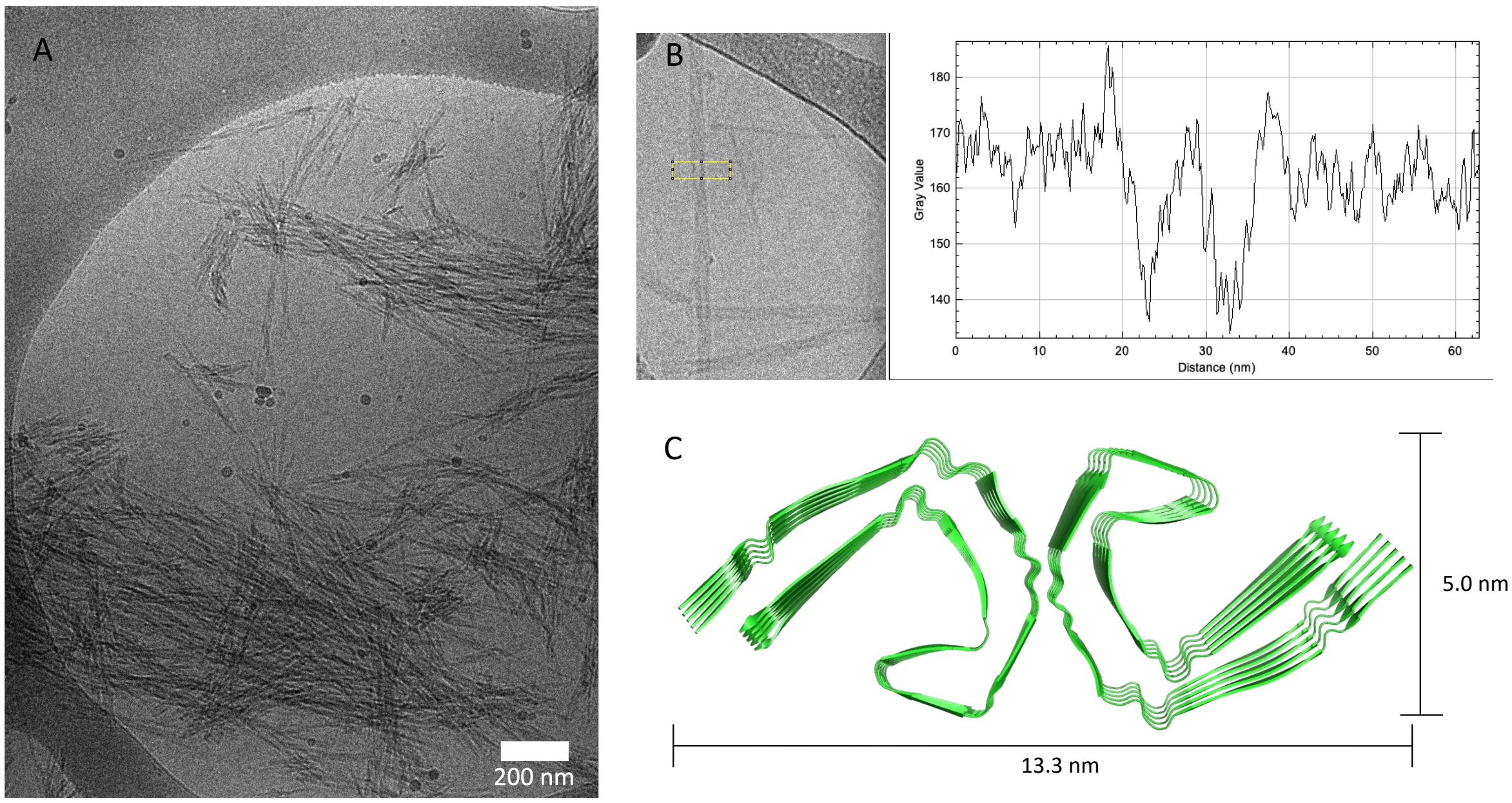

Figure S2: Cryo-EM image of seeds used for the aggregation kinetics, prepared by incubating 14 µM tau at 37 °C with magnetic stirring bar over night. **A**: The average length of the fibril seeds was estimated from cryo-EM imaging. A total of 25 fibrils with distinguishable lengths from several images across multiple grid squares were measured using ImageJ, yielding an average length of 450±200 nm. **B**: Grey-value profile of a representative fibril. The fibril seems to compose two approximately 6 nm thick protofilaments. This is in agreement with the cross-sectional diameter of the ordered part of a tau molecule in one protofilament [3], as seen in **C** (PDB: 5O3L). Thus, each fibril seems to have two monomers per plane.

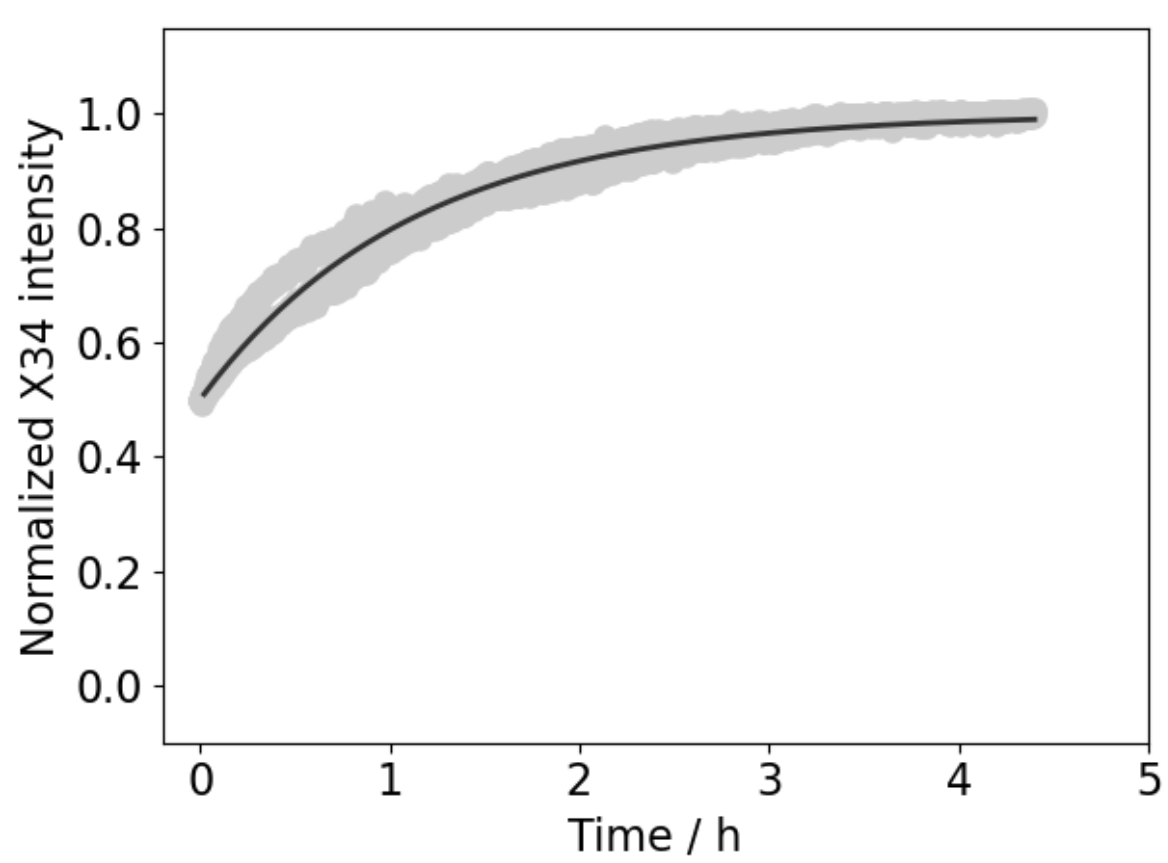

Figure S3: Fit of tau kinetics data with 50 % seeds (no JB6) with an exponential function on the form $F(t) = A(1 - Be^{-kt})$. A is set to 1 and B to 0.5, according to the end and start fraction of fibrils, respectively. The good description of the data is consistent with that fibril formation is highly dominated by elongation at 50 % seeds, which is why this situation is suitable for examining elongation rate inhibition.

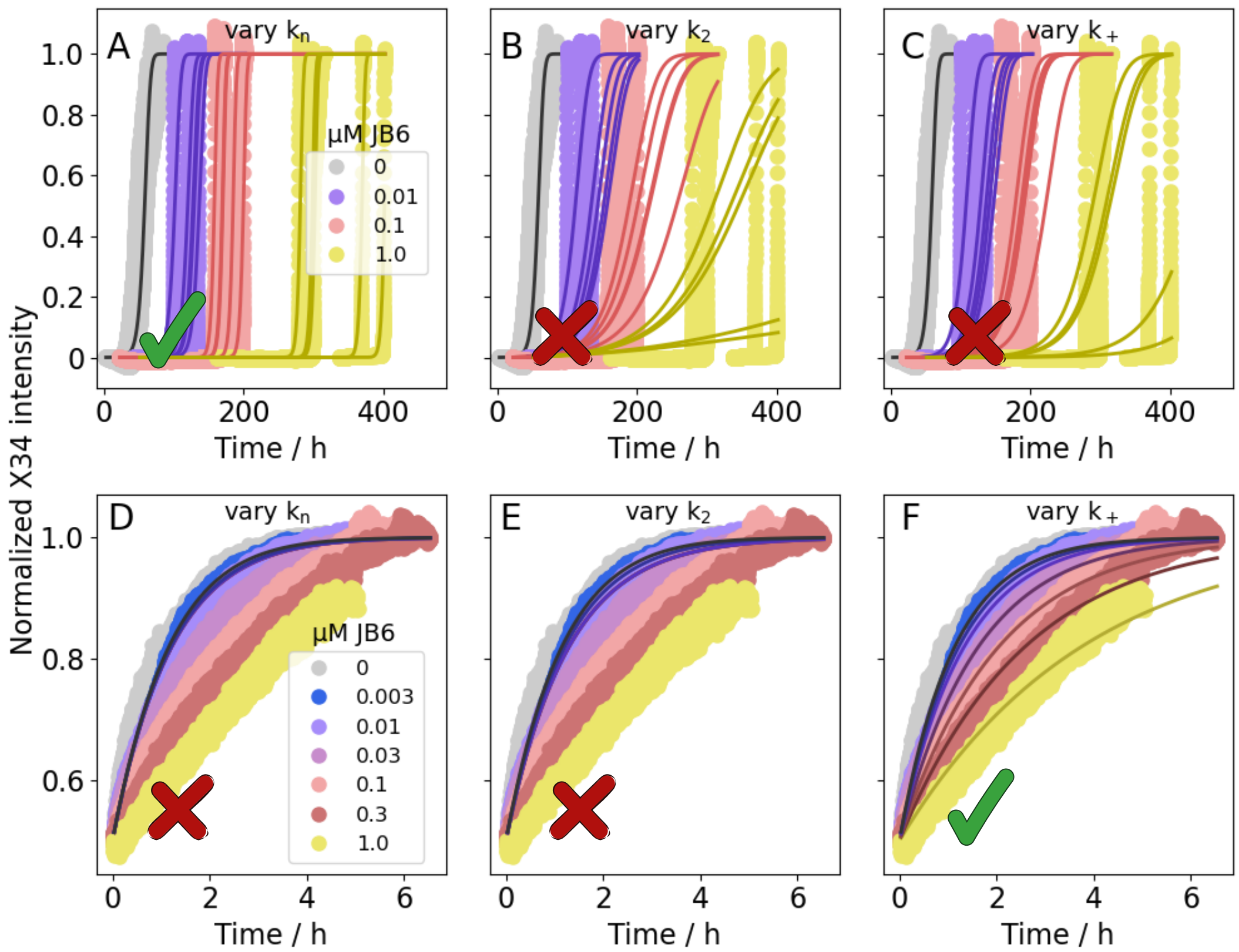

Figure S4: Fits to 0 % seeds in **A-C** and 50 % seeds in **D-F**, in each case using two of the rate constants of tau alone as fixed parameters and one rate constant as a variable parameter, as indicated at the top of each panel.

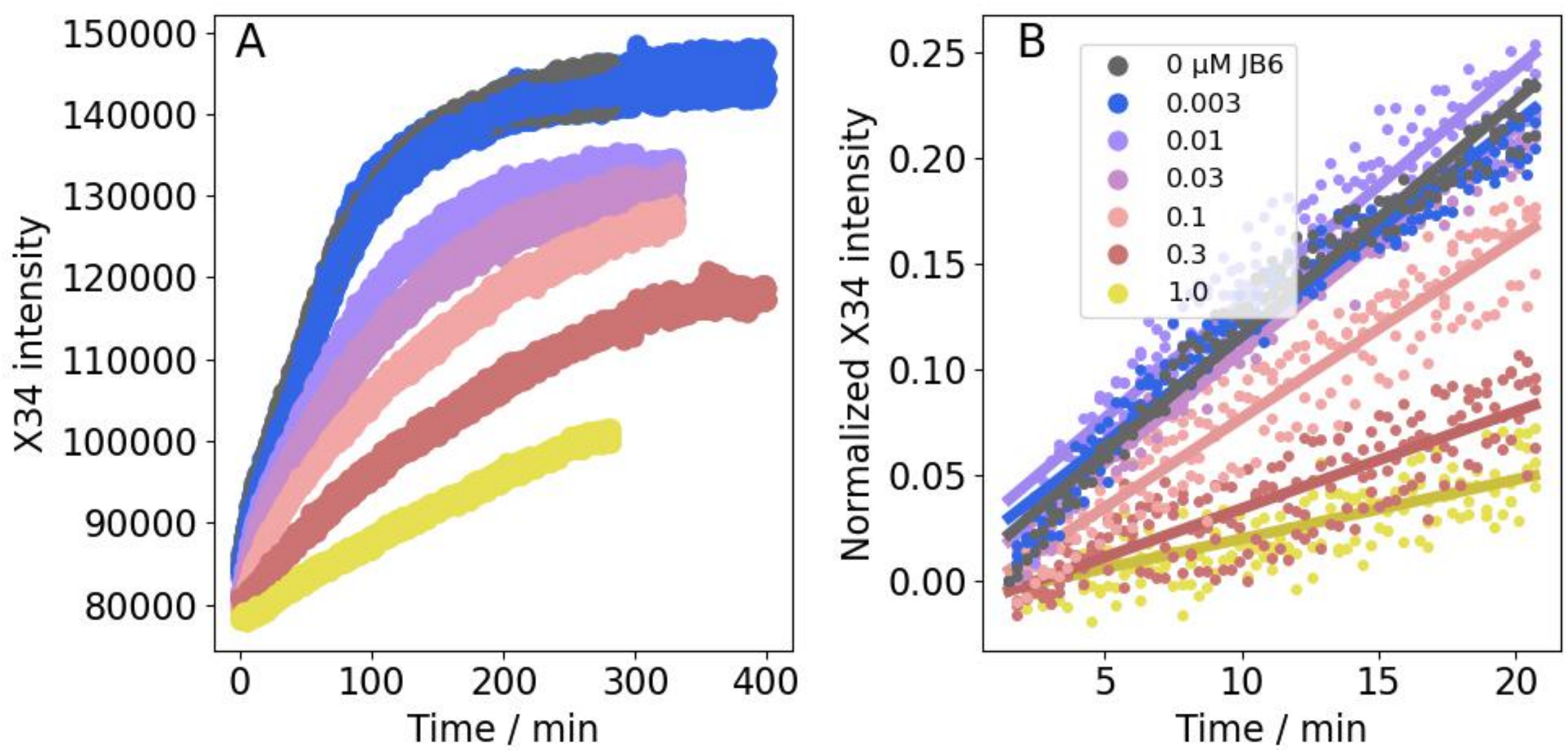

Figure S5: An alternative way of analyzing the change in elongation rate constants. Kinetic traces following 3.3 µM tau monomers aggregating, in the presence of 3.3 µM seeds and varying concentrations JB6, in triplicates. **A**: Raw data of X34 intensities, as a reporter on fibril mass. **B**: Analysis of the initial slopes. Shown are the first 20 minutes of the normalized traces (using starting points and plateaus) and linear regressions of the time interval.

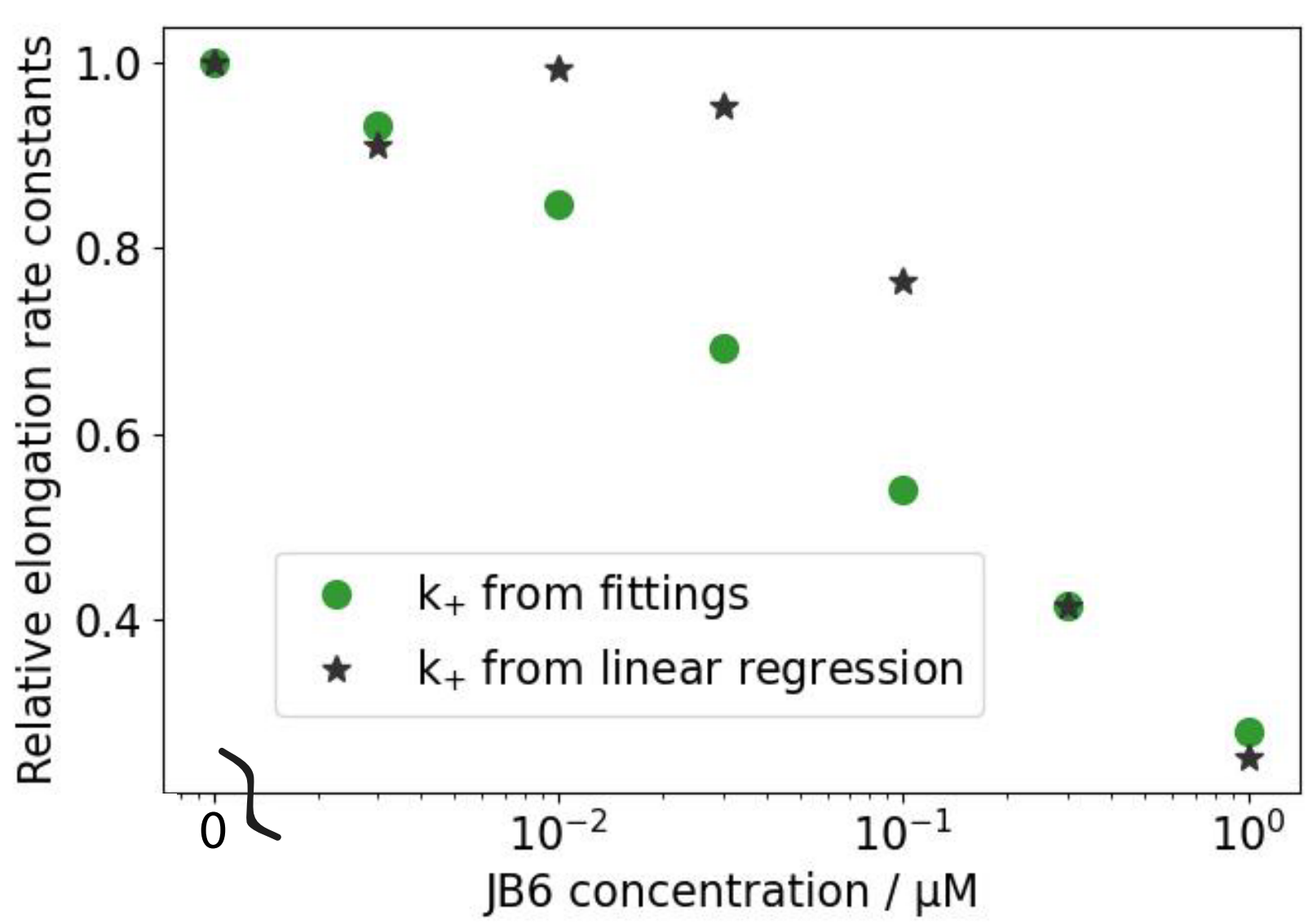

Figure S6: Comparing two different ways to obtain the how JB6 affects the elongation rate constants, relative to tau alone. The fits from Figure 3D correspond to the green circles, and the result from the initial slopes in Figure S5 provide with the black stars.

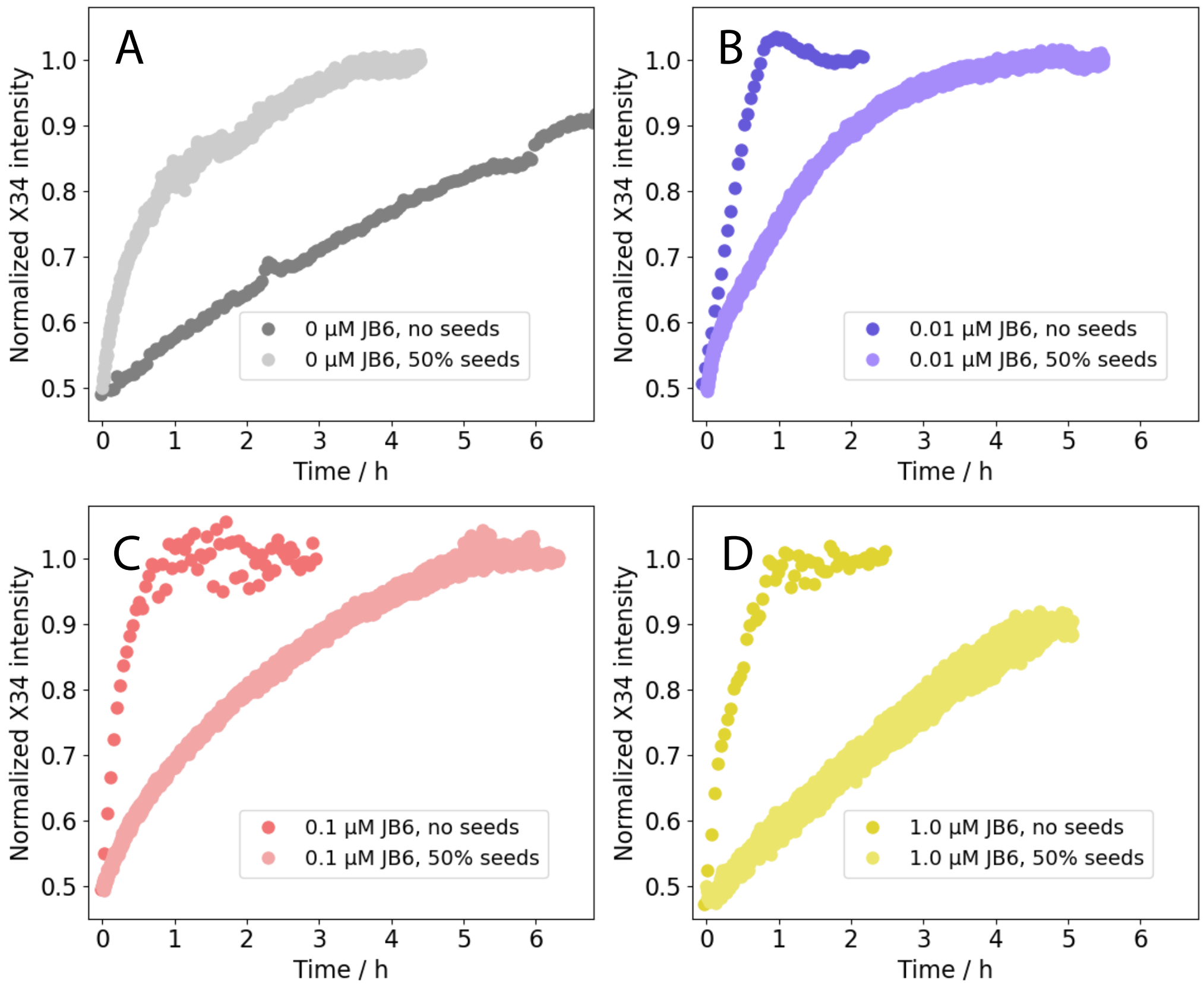

Figure S7: Comparison of the curve shapes for the last 50 % of the kinetic traces with no seeds and 50 % seeds, at different JB6 concentrations. **A**: no JB6. **B**: 0.01 µM JB6. **C**: 0.1 µM JB6. **D**: 1 µM JB6. Common for all cases with JB6 present is that the slope without seeds is steeper. For tau alone, the non-seeded reaction is slower, which is likely due to the difference in length of the 50 % fibrils present at time 0 in the graph (which is the $t_{1/2}$ for the non-seeded reaction. The seeds where prepared with stirring, which result in shorter fibrils compared to the ones formed in the non-shaking plate.

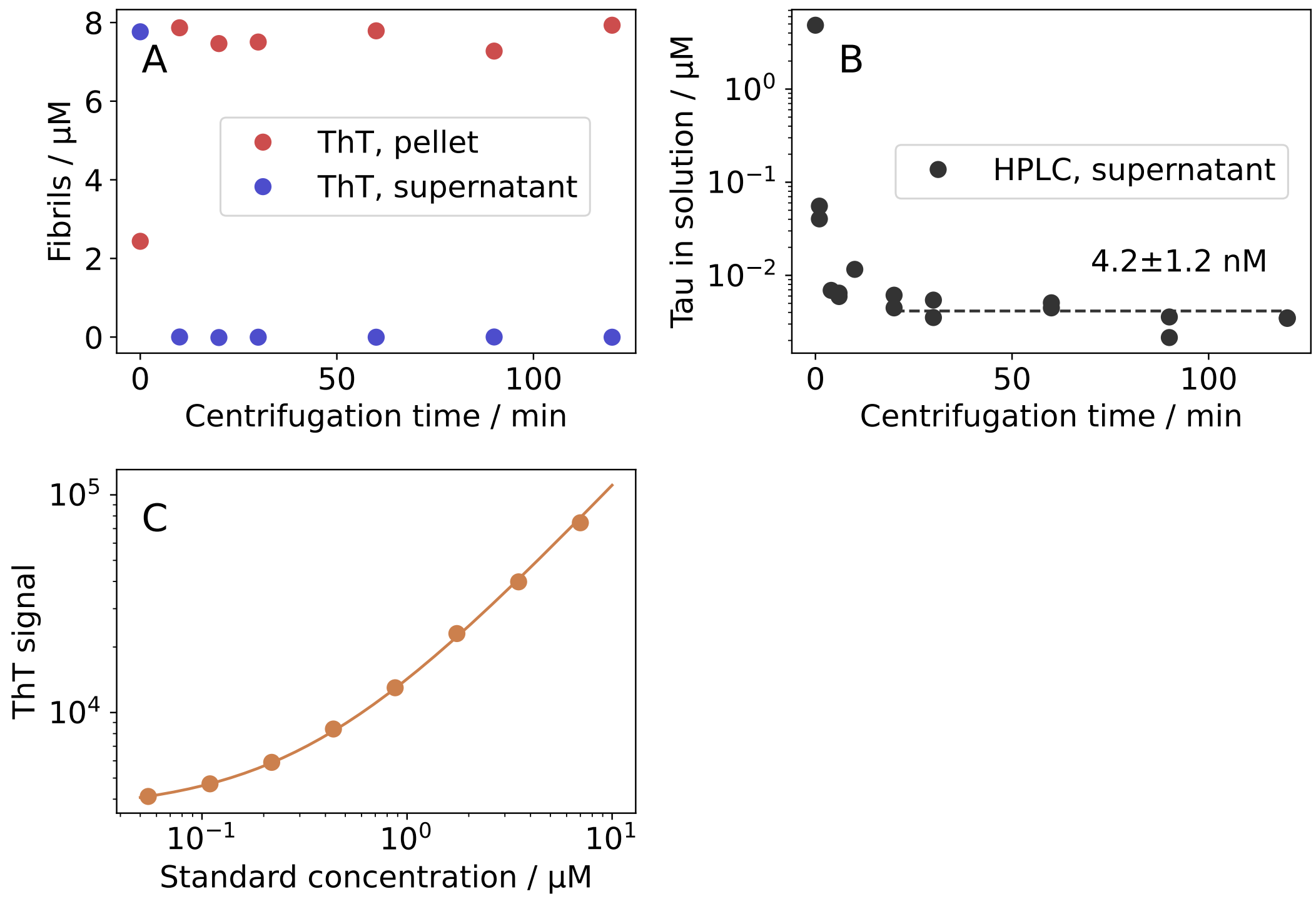

Figure S8: Pelleting of tau as a function of centrifugation time at 20 000 g, probed with florescence in **A** and HPLC in **B**. To convert the fluorescence signal to concentration of fibrils, a standard curve with fibrils at different concentrations was run simultaneously. Already after a few minutes most of the fibrils had sedimented, and after ∼ 20 min the measurements are robust with a soluble tau concentration of 4.2±1.2 nM. **C**: Standard curve of ThT signal of tau fibrils, used to convert the ThT signal in **A** to fibril concentration.

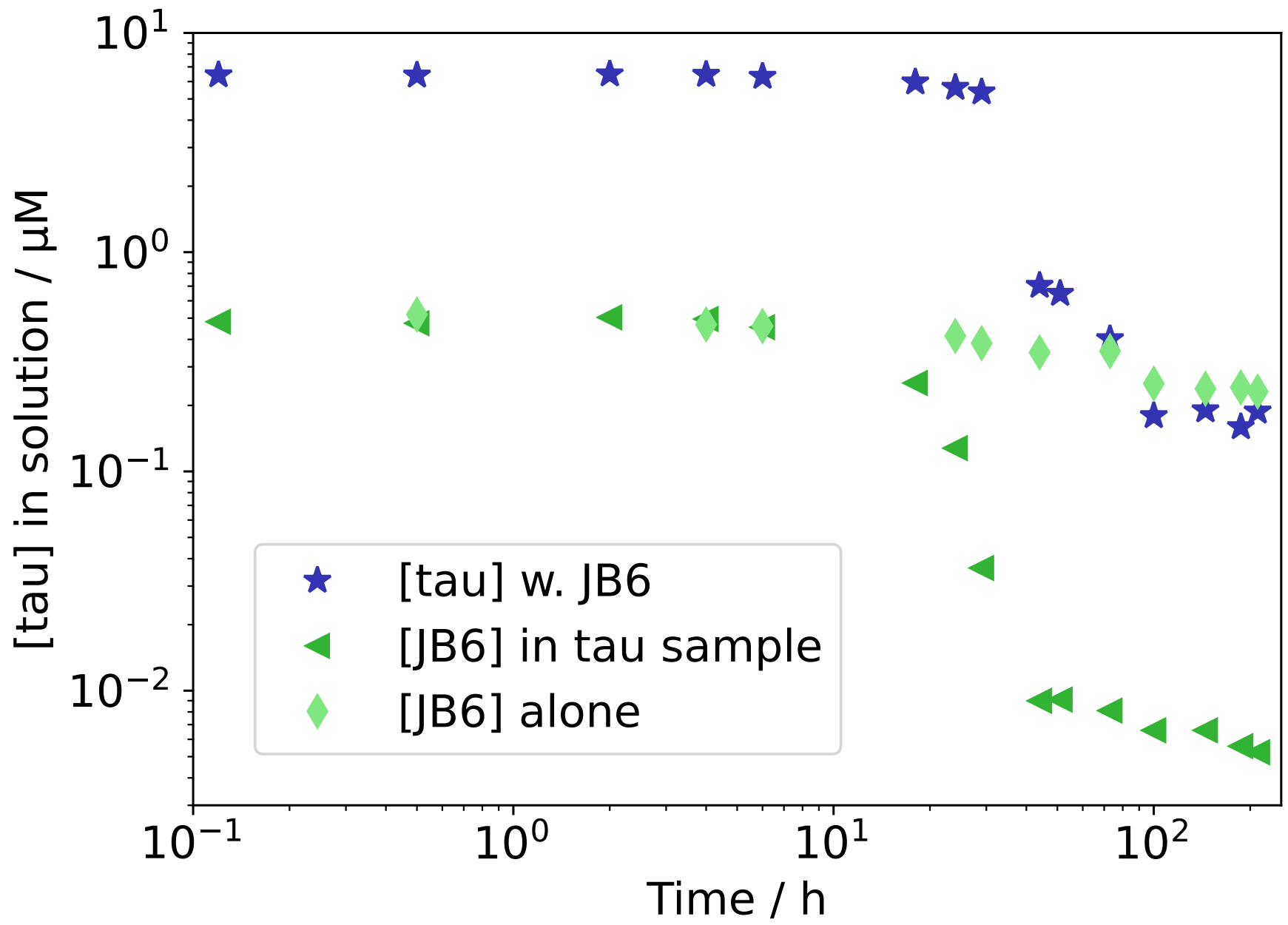

Figure S9: HPLC UV absorbance quantification to probe how the concentration of soluble JB6 changes on its own with time, compared to how the concentrations of tau and JB6 changes during the aggregation process, as shown in Figure 5. All samples were treated in the same way.

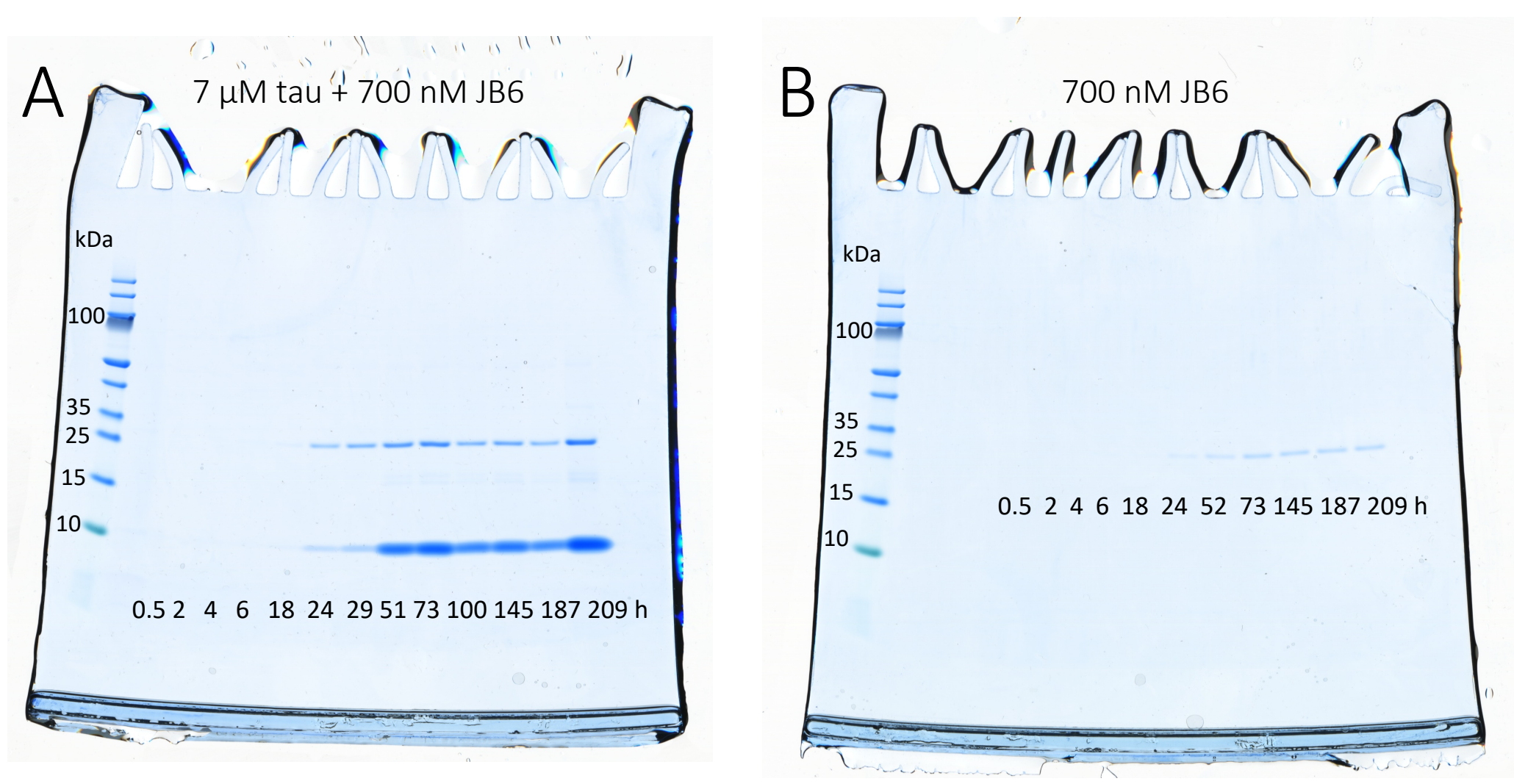

Figure S10: SDS PAGE on the pellets of the same samples as were measured with HPLC (Figure S9. **A**: 7 μM + 700 nM JB6. **B**: 700 nM JB6 only. The gels were run with electrophoresis in parallel and stained and de-stained with Coomassie blue for equally long times.

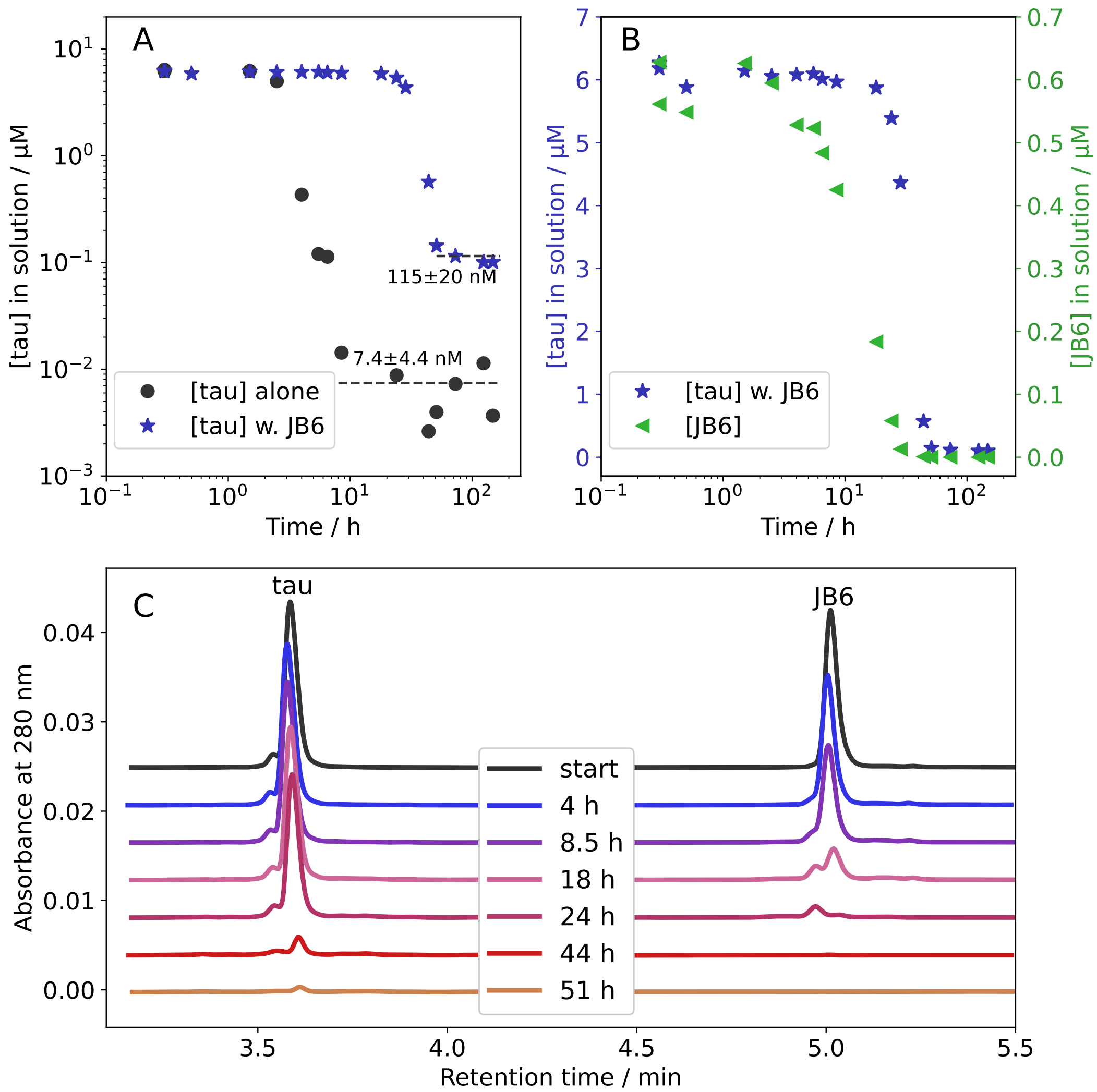

Figure S11: Similar to Figure 5, but performed at pH 8.0 and without 150 mM NaCl. The concentrations of tau and JB6 are simultaneously measured using UV-absorbance in reversed phase HPLC, during the aggregation process. Prior to measurements, fibrils were removed by centrifugation. **A**: The concentration of soluble tau during the ongoing aggregation reaction, with and without the presence of JB6. Shown are the mean values and standard deviations of the last 7 and 4 time points, for tau alone and tau with JB6, respectively. **B**: For the sample with both tau and JB6, the concentrations of tau and JB6 in solution are plotted using separate linear y-axes. **C**: The absorbance chromatograms at 280 nm of the supernatant from the sample with both tau and JB6, where the peaks are integrated to obtain the data in **A-B**.

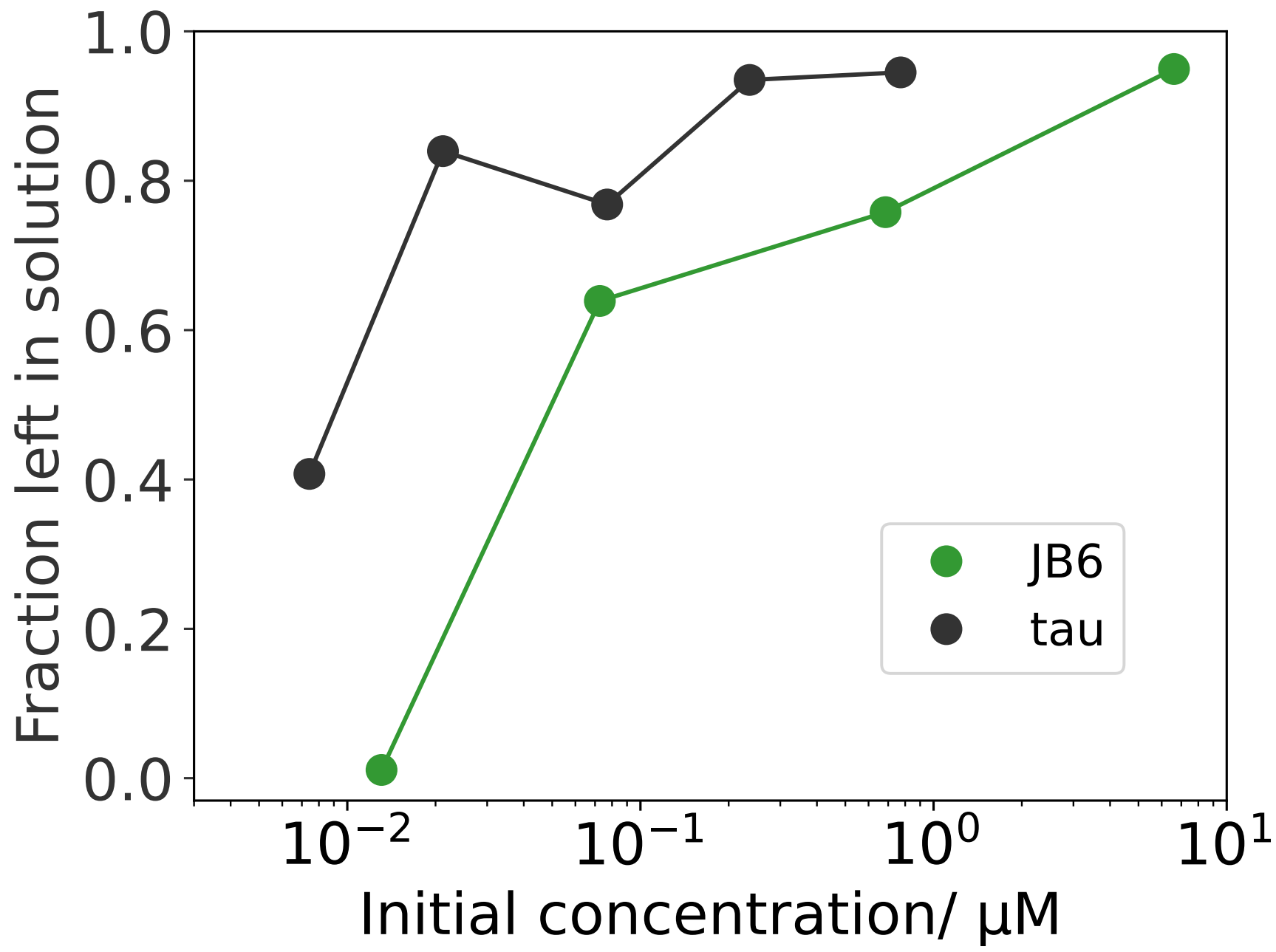

Figure S12: Surface adsorption of tau and JB6 in the experimental setup used in Figure 5. Separate samples with tau respectively JB6 was prepared at different concentrations and measured with HPLC UV absorbance directly after dilution from the protein stock. After the sample had been subjected to the same steps as when separating fibrils and monomers, the concentrations were measured again. The ratio of initial and final concentration is given as the "fraction left in solution", plotted versus the initial concentration.

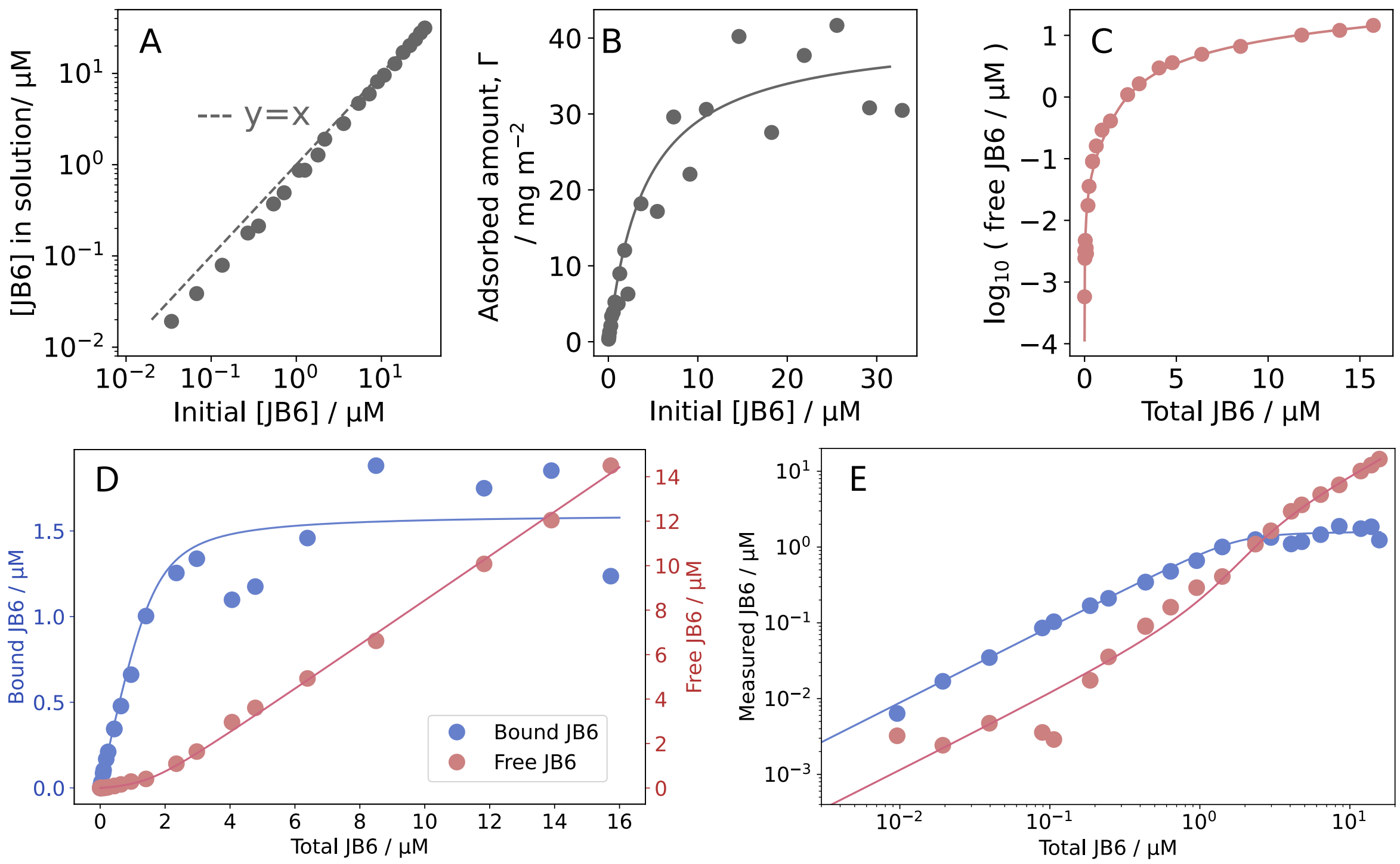

Figure S13: **A:** Measured concentrations of JB6 in each tube after incubation in the tube, before tau fibrils were added, in a log-log plot to see the deviation from a 1:1 ratio at low concentrations (ascribed to surface adsorption). **B**: Adsorption isotherm from the data in A, with a fit to Equation 1. **C:** JB6 binding to fibrils. K_D determination with log10 transformed free JB6 concentration. K_D is here fitted to 205 nM, with the saturation stoichiometry parameter, d, fixed to 1.6 from the fitting of linear values. **D**: The complete data series from Figure 6C, with separate y-axis. **E**: Same data as in D, but with a common, logarithmic y-axis.

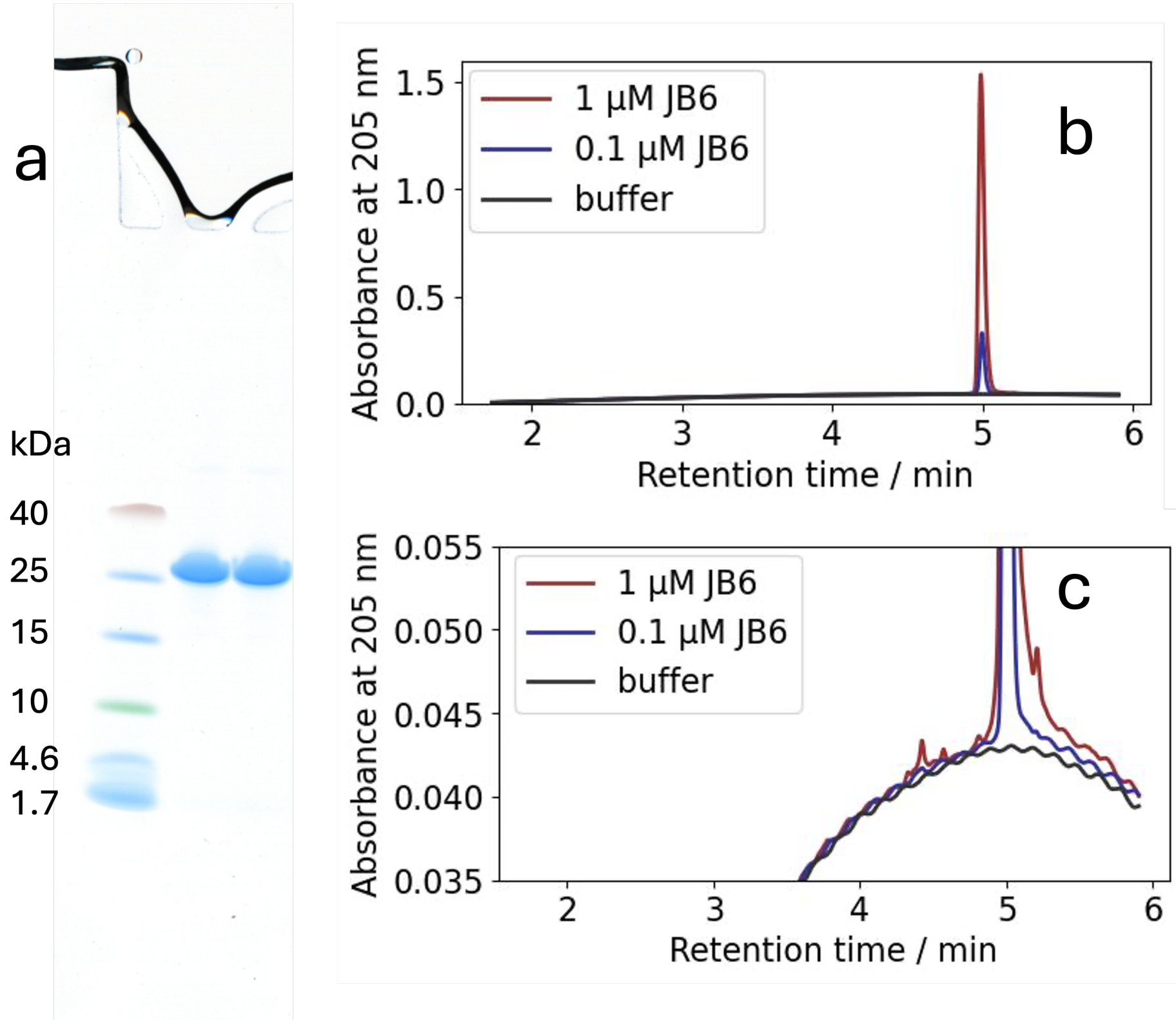

Figure S14: Purity estimation of JB6 **A**: SDS PAGE of 30 μM JB6 in both lanes. **B**: HPLC chromatograms from reversed phase, absorbance at 205 nm of 0.1 and 1 μM JB6. **C**: Zoom-in close to the baseline to estimate the size of eventual impurities. The small peaks have each an area corresponding to less than 0.1 % of the JB6 peak.

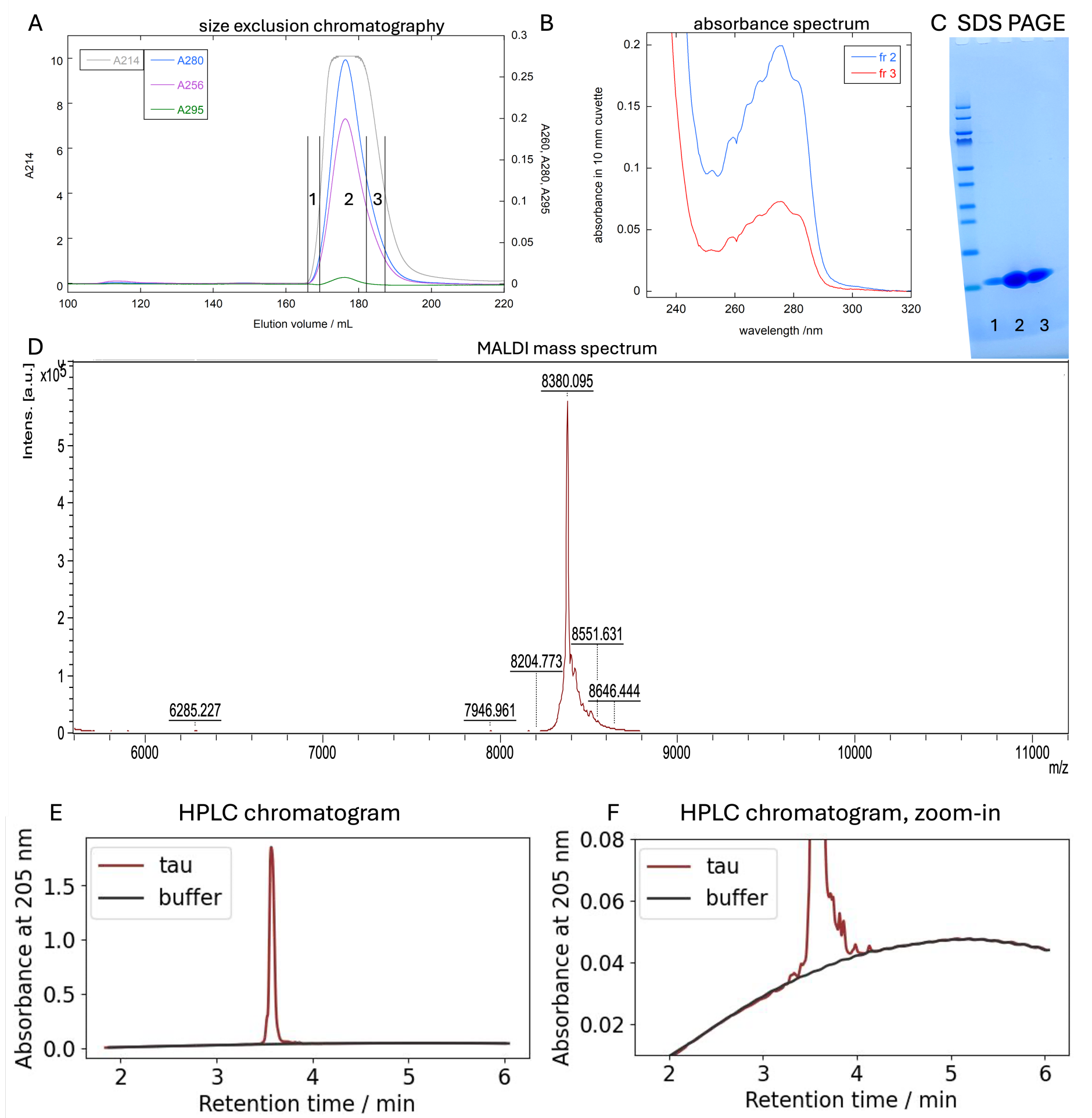

Figure S15: Last step of tau purification and validation of identity and purity. **A**: Final large scale SEC on a 26/600 Superdex75 column in 20 mM NaP, 0.2 mM EDTA, pH 8.0. **B** Absorbance spectra in 10 mm cuvette of collected fractions. **C**: SDS PAGE of collected fractions on a 10-20 % Tris-Tricine gel (Novex). **D** MALDI mass spectrum of the main fraction. Fractions 2 and 3 were pooled and aliquoted, lyophylized and stored at -20 °C. **E**: HPLC chromatograms from reversed phase, absorbance at 205 nm of 10 µM tau. **F**: Zoom-in close to the baseline to estimate the size of eventual impurities. The small peaks have each an area corresponding to about 0.1 % of the tau peak.

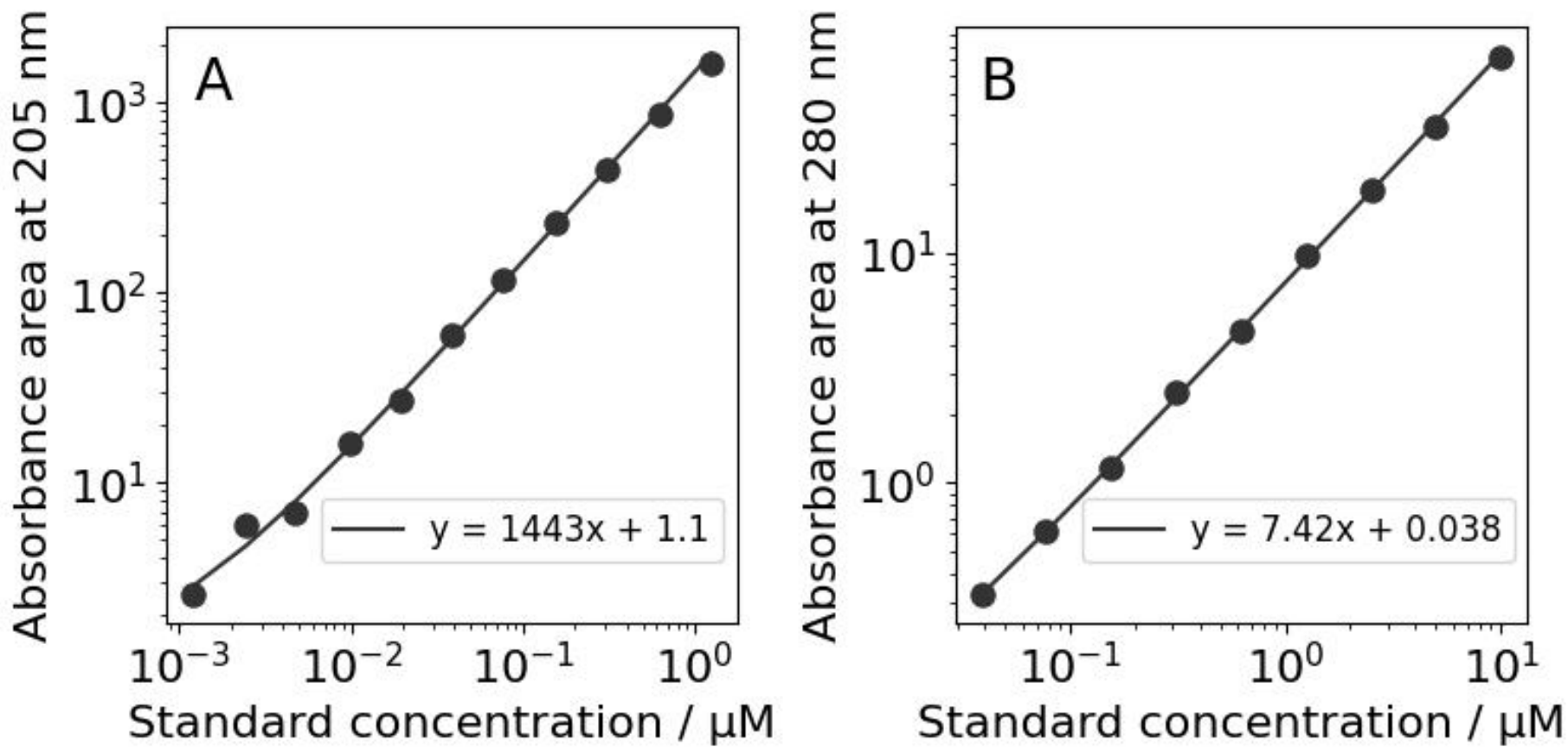

Figure S16: Standard curves for tau with HPLC UV absorption, with linear fits, weighted with concentration^{-1}. Absorbance at 205 nm in **A** and at 280 nm in **B**. With this methodology, the standard curve accurately determines tau concentrations between 1 nM and 10 µM.

References

[1] Carlsson, A., Olsson, U. & Linse, S. On the micelle formation of DNAJB6b. *QRB Discovery* **4**, e6 (2023). URL `https://www-cambridge-org.ludwig.lub.lu.se/core/journals/qrb-discovery/article/on-the-micelle-formation-of-dnajb6b/4E97ACD501AAA6516BBAF314EE4E3F1A`.

[2] Pallbo, J., Fornasier, M., Linse, S. & Olsson, U. AirWater Interfacial Adsorption of the Chaperone Protein DNAJB6b. *Langmuir* **41**, 19146–19155 (2025).

[3] Fitzpatrick, A. W. *et al.* Cryo-EM structures of tau filaments from Alzheimers disease. *Nature 2017 547:7662* **547**, 185–190 (2017). URL `https://www-nature-com.ludwig.lub.lu.se/articles/nature23002`.
