# Supplementary material for: Human chaperone DNAJB6b suppresses tau fibril formation through co-aggregation
Source: Commun Chem. 2026 Aug 1;9:263. doi: 10.1038/s42004-026-02108-1 (PMC13434469; doi:10.1038/s42004-026-02108-1)
Supplement: Supplementary file 2 [file 42004_2026_2108_MOESM2_ESM.pdf]

Description of Additional Supplementary Files:

File: Supplementary Data 1

Description: NMR Spectra
