# Supplementary material for: Human chaperone DNAJB6b suppresses tau fibril formation through co-aggregation
Source: Commun Chem. 2026 Aug 1;9:263. doi: 10.1038/s42004-026-02108-1 (PMC13434469; doi:10.1038/s42004-026-02108-1)
Supplement: Supplementary file 3 [file 42004_2026_2108_MOESM3_ESM.pdf]

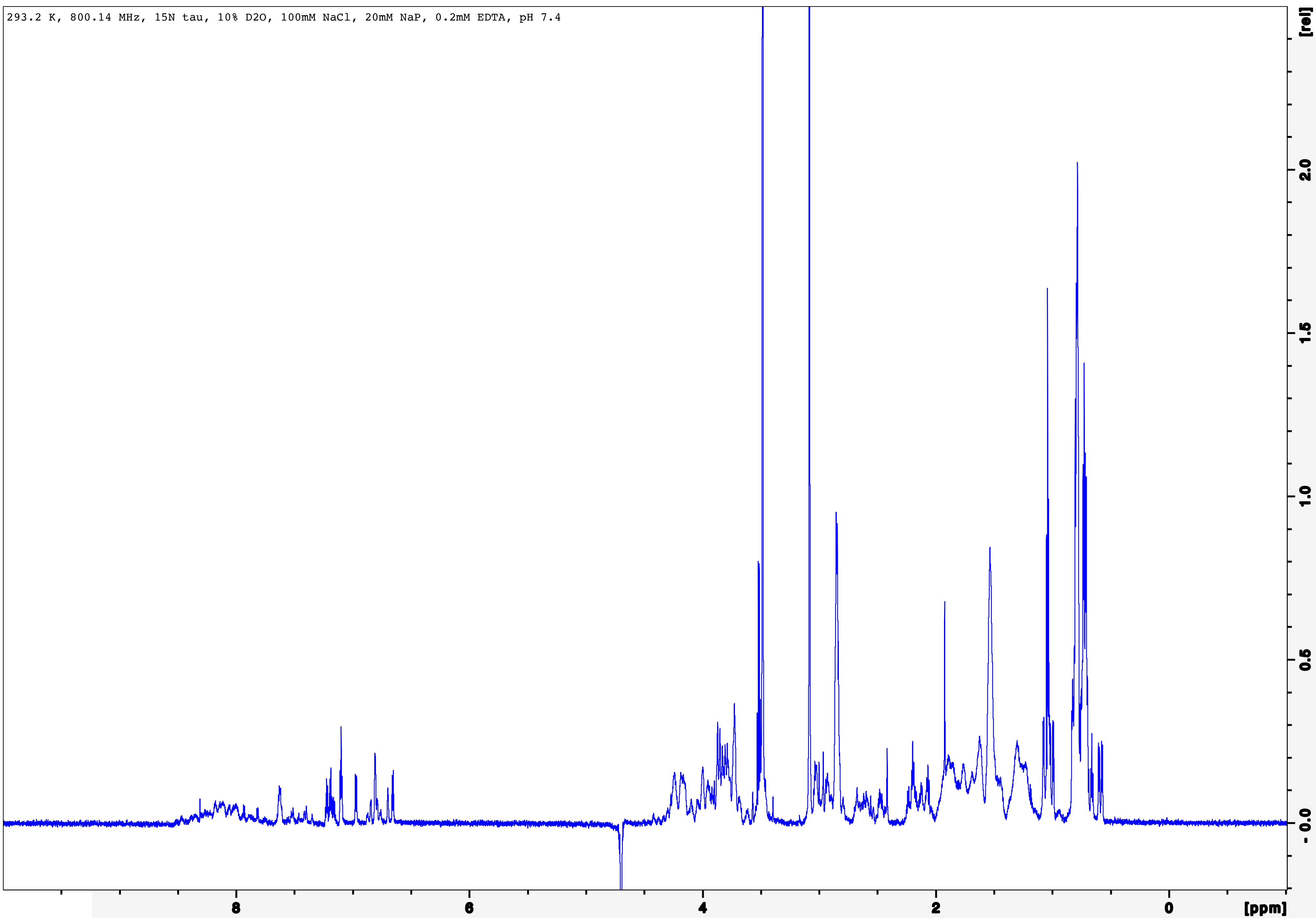
293.2 K, 800.14 MHz, 15N tau, 10% D2O, 100mM NaCl, 20mM NaP, 0.2mM EDTA, pH 7.4
[rel]
2.0
1.5
1.0
0.5
- 0.0
8
6
4
2
0
[ppm]

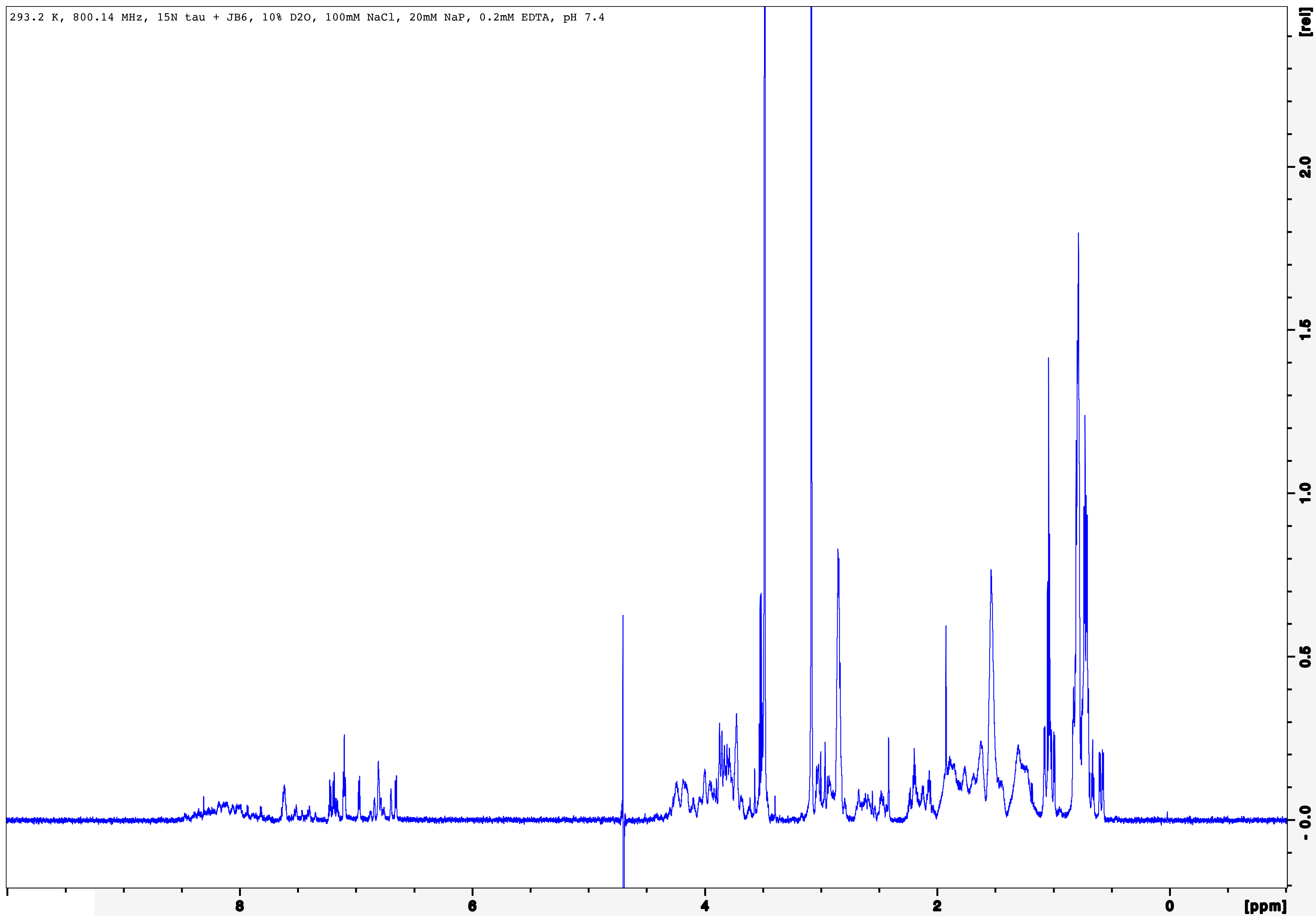
293.2 K, 800.14 MHz, 15N tau + JB6, 10% D2O, 100mM NaCl, 20mM NaP, 0.2mM EDTA, pH 7.4
[rel]
2.0
1.5
1.0
0.5
- 0.0
8
6
4
2
0
[ppm]

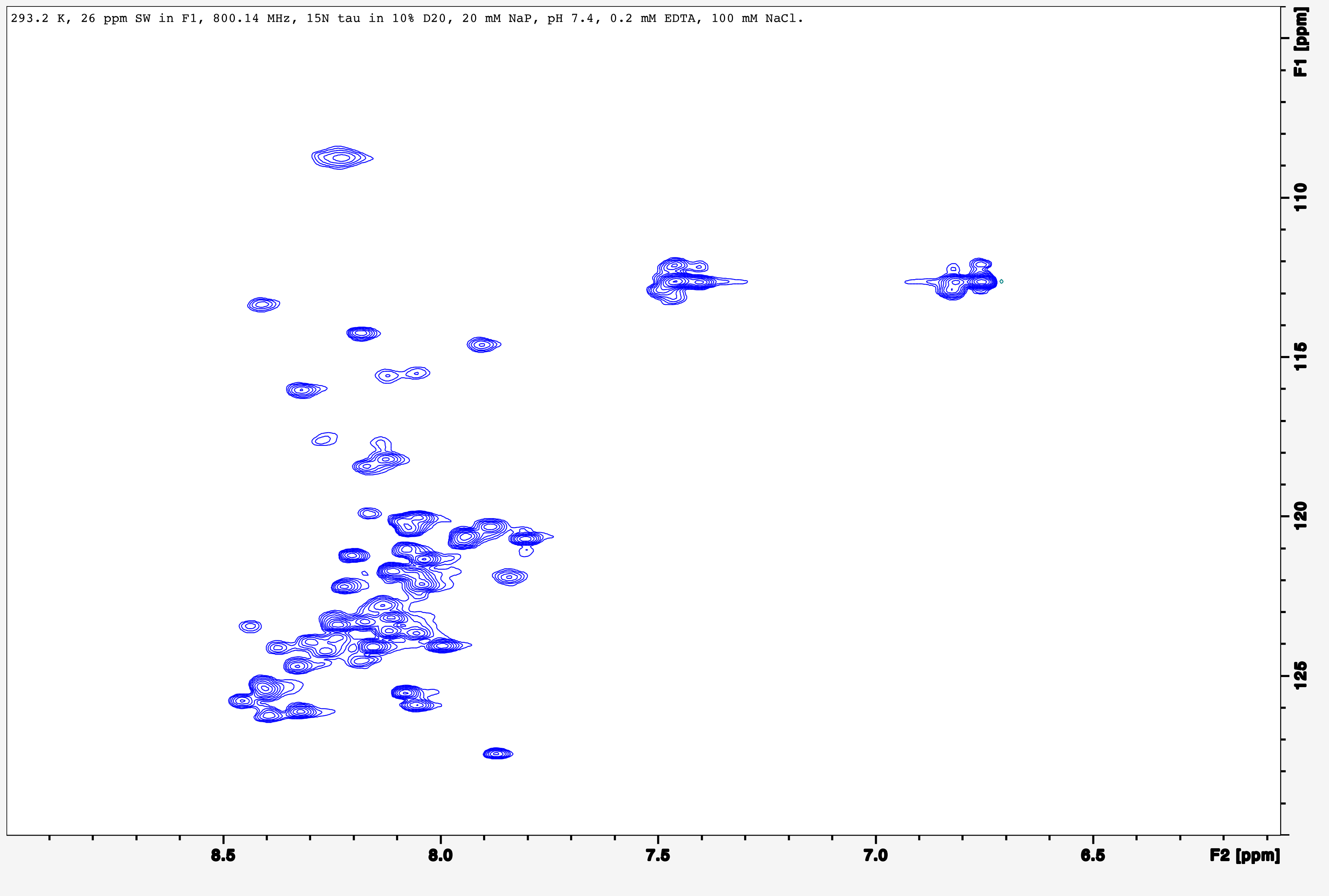
293.2 K, 26 ppm SW in F1, 800.14 MHz, 15N tau in 10% D20, 20 mM NaP, pH 7.4, 0.2 mM EDTA, 100 mM NaCl.
F1 [ppm]
110
115
120
125
8.5
8.0
7.5
7.0
6.5
F2 [ppm]

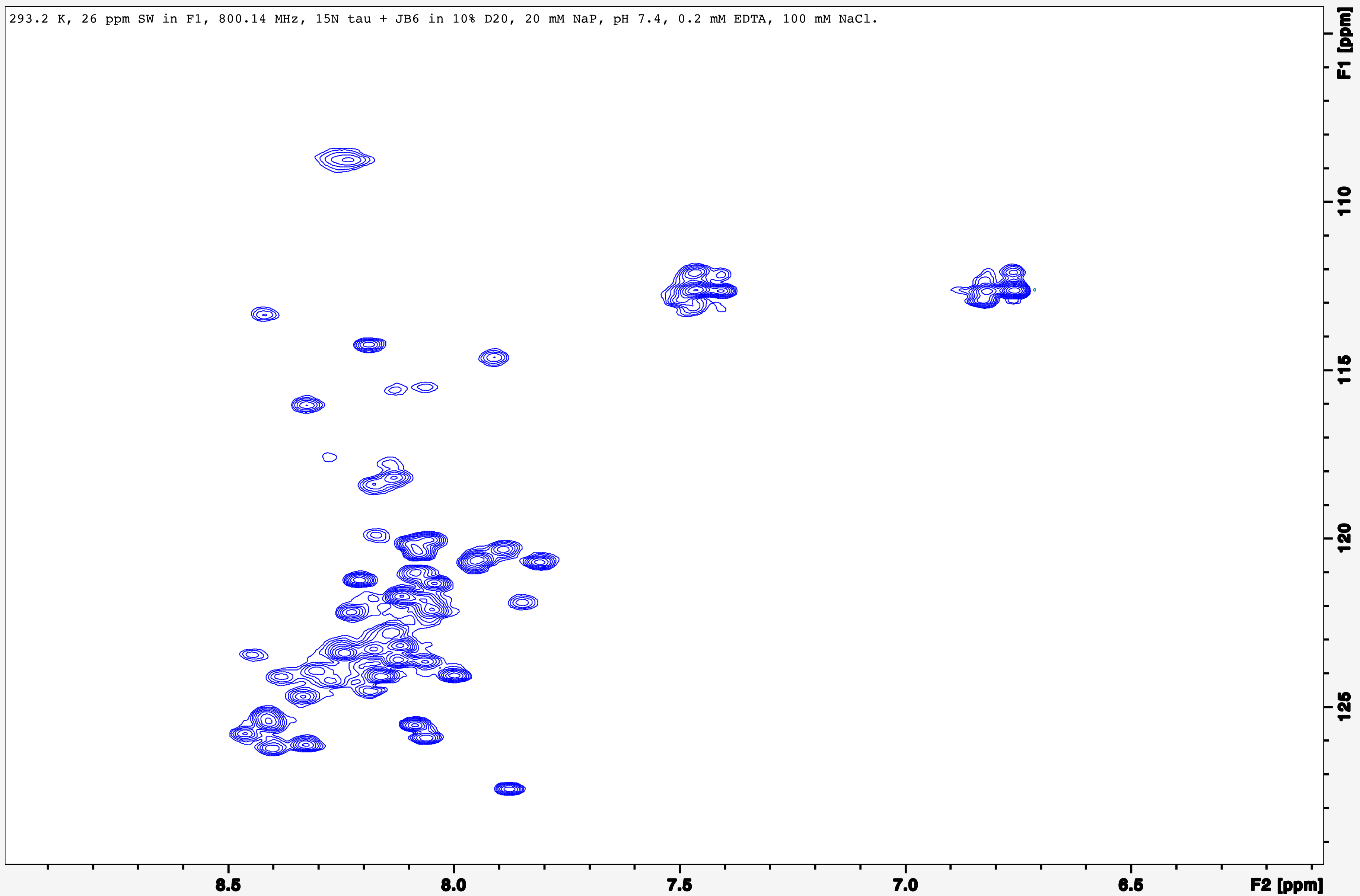
293.2 K, 26 ppm SW in F1, 800.14 MHz, 15N tau + JB6 in 10% D2O, 20 mM NaP, pH 7.4, 0.2 mM EDTA, 100 mM NaCl.
F1 [ppm]
110
115
120
125
8.5
8.0
7.5
7.0
6.5
F2 [ppm]
